# Schooling Trajectories and the Development of Brain Dynamics: A Comparative Study of Montessori and Traditional Education

**DOI:** 10.1002/advs.202524343

**Published:** 2026-04-20

**Authors:** Elvira del Agua, Anira Escrichs, Yonatan Sanz Perl, Morten L. Kringelbach, Adele Diamond, Solange Denervaud, Gustavo Deco

**Affiliations:** ^1^ Center for Brain and Cognition Computational Neuroscience Group Department of Information and Communication Technologies Universitat Pompeu Fabra Barcelona Spain; ^2^ Departamento de Matemática y Ciencias Universidad de San Andrés Buenos Aires Argentina; ^3^ National Scientific and Technical Research Council (CONICET) CABA Buenos Aires Argentina; ^4^ Center for Music in the Brain Department of Clinical Medicine Aarhus University Denmark; ^5^ Centre for Eudaimonia and Human Flourishing Linacre College University of Oxford Oxford UK; ^6^ Department of Psychiatry University of Oxford Oxford UK; ^7^ Program in Developmental Cognitive Neuroscience Department of Psychiatry University of British Columbia Vancouver British Columbia Canada; ^8^ CIBM Center for Biomedical Imaging Lausanne Switzerland; ^9^ MRI Imaging and Technology, Polytechnical School of Lausanne Swiss Federal Institute of Technology Lausanne (EPFL) Lausanne Switzerland; ^10^ Department of Radiology Lausanne University Hospital and University of Lausanne (CHUV‐UNIL) Lausanne Switzerland; ^11^ Institució Catalana de la Recerca i Estudis Avançats (ICREA) Barcelona Spain; ^12^ Department of Psychology & Centre for Cognitive Neuroscience Paris‐Lodron‐University Salzburg Austria

**Keywords:** childhood neurodevelopment, computational neuroscience, pedagogy, resting‐state fMRI, whole‐brain dynamics

## Abstract

Learning environments may shape both children's cognitive outcomes and the maturation of large‐scale neural dynamics. We investigate whether pedagogical context modulates brain activity and cognition in 96 students (4–15 years) enrolled in Montessori (MSC) or traditional (TSC) schools. Montessori emphasizes self‐directed exploration with increasing independence, whereas traditional classrooms shift from play‐based early learning to teacher‐directed instruction. To examine how neural development diverges across educational trajectories, we quantify the temporal asymmetry (“non‐reversibility”) of fMRI signals during resting state and movie‐watching. Behaviorally, MSC students outperform TSC peers in language, mathematics, and both divergent and convergent thinking. Neural patterns diverge with age: during rest, non‐reversibility increases in MSC but decreases in TSC, primarily in sensorimotor, dorsal attention, and frontoparietal networks; during movie‐watching, differences are strongest in younger children, especially in subcortical, visual, and default mode networks. Exploratory analyses reveal higher non‐reversibility in females, with larger sex differences in TSC. These findings suggest that pedagogical context may shape developmental trajectories and variability of neural dynamics. Montessori education may foster more adaptive brain dynamics, support flexible and creative thinking, and reduce gender disparities. Longitudinal research is needed to clarify what non‐reversibility reflects and how educational approaches sculpt brain development.

## Graphical abstract

We investigate whether Montessori and traditional schooling systems shape the developmental trajectory of large‐scale brain dynamics in different ways. We quantify the arrow of time (“non‐reversibility”) in neural activity during resting state and movie‐watching, revealing distinct maturational patterns. These findings suggest that pedagogical context may shape developmental trajectories and variability of neural dynamics.

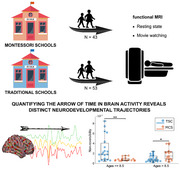

## 1 Introduction

Characteristics of a school's philosophy about what an educational environment should be like, how learning occurs, and the teaching practices that best foster learning play a critical role in shaping learning outcomes and socio‐emotional development [1, 2, 3]. The balance a teacher establishes between guidance and autonomy and the consistency of the teacher's expectations forms a scaffold through which students engage with both their learning context and its content [4, 5, 6]. Emerging research in education highlights that the most effective pedagogical models are not those that impose structure or permit unbounded freedom, but rather those that provide a consistent and coherent framework while progressively supporting increasing autonomy aligned with developmental needs; the benefits of guided vs. self‐directed learning are likely age‐dependent [7, 8, 9].

These principles map onto well‐characterized neurodevelopmental changes. Early childhood features refinement of sensory and motor systems and the emergence of executive functions (including inhibitory control, working memory, and cognitive flexibility). Across middle childhood, cognition progressively shifts from more concrete, hands‐on modes of thinking toward more abstract, complex forms of reasoning. This change is supported by the increasing hierarchical functional organization of large‐scale brain networks [10, 11, 12, 13], marked by a transition from diffuse to more focal recruitment of cortical regions [14] and greater segregation among higher‐order systems [15]. This period sees the development of richer capacity to generate own strategies for learning and behaving, constructing internal guidelines rather than relying solely on external instruction. It is also when perspective‐taking, as well as autonomous problem solving, becomes more robust, and corresponds to the so‐called “age of reason” in developmental psychology [15]. These neurodevelopmental milestones suggest that optimal pedagogical strategies should probably dynamically evolve alongside the child, perhaps beginning with structured, guided learning and then gradually shifting toward more self‐directed exploration and less structured guidance.

Montessori education provides a compelling model of developmentally aligned instruction [16, 17], as it was designed around the principles of sensitive periods and developmental readiness. From ages 3 to 6 (i.e., primary level), children are introduced to a carefully curated set of tactile didactic materials. The teacher presents these materials, often to a small group of children at the same time, and demonstrates their proper use in a deliberate and structured manner. Following the presentation, children are invited to engage with the materials independently, choosing tasks according to their interests within a thoughtfully prepared and orderly environment that supports concentration, autonomy, and intrinsic motivation. After age 6 (i.e., elementary level), instruction increasingly incorporates peer‐led inquiry, interdisciplinary exploration, and project‐based learning [16, 18]. This structured yet flexible progression, from scaffolded development of self‐regulation to increasingly autonomous and collaborative learning, closely mirrors known patterns of functional brain segregation followed by integration [14, 19]. In contrast, traditional schooling systems in Switzerland typically move from open‐ended, free play, exploratory experiences in early childhood to more rigid, adult‐led instruction during elementary years in preparation for standardized examinations, potentially reducing opportunities for curiosity‐driven and socially enriching collaborative learning.

Environmentally shaped plasticity, the brain's ability to reorganize in response to an individual's experience, is particularly pronounced during childhood [20, 21]. Accordingly, early childhood curricula that support autonomy, learning by doing, and child‐initiated learning, such as those found in Montessori and “Tools of the Mind” classrooms, have been shown to support the development of executive functions, such as working memory, cognitive flexibility, and inhibitory control [1, 3], capacities that are foundational for later academic success [22, 23, 24, 25]. As students mature and become increasingly capable of self‐regulation and engaging with more abstract content (i.e., reflecting the integration of broader functional networks), learning environments that restrict autonomy and emphasize conformity may dampen neural development in regions supporting executive control, decision‐making, and attention, simply because students have fewer opportunities to practice and refine these skills. Conversely, less structured environments across elementary years that encourage exploration and creativity might promote continued strengthening of neural pathways underlying cognitive flexibility and problem‐solving [26, 27, 28], as students must actively deploy and refine these abilities. Consistent with this perspective, research comparing Montessori‐schooled children (MSC) with traditionally schooled children (TSC) has shown the benefits of the Montessori method for academic outcomes, creative abilities, socio‐emotional skills, executive functions, and self‐direction [3, 29]. Together, these findings suggest that pedagogical context may partially shape the organization and maturation of large‐scale neural systems in ways that support adaptive cognitive development.

Here, we looked at the effects of pedagogical context (MSC vs. TSC) on cognitive and affective outcomes and whether pedagogical context appears to affect age‐dependent properties of large‐scale brain dynamics. It is the first study we know of to investigate the effects of pedagogical practice or philosophy using a non‐causal framework for functional dynamics of brain networks across development. We studied 96 students aged 4 to 16 years who were consistently schooled in either Montessori or traditional systems. We anchored analyses at age 8.5, as it marks an inflexion point when more structured instruction should start to give way to more autonomy in the learning process and more collaborative learning, according to the development of intrinsic neural dynamics. We compared young (4.6–8.4 years old) and older (8.5–15.2 years old) participants using 3‐min resting state runs (undirected cognition), as well as 3‐min movie‐watching data (externally guided attention) using functional magnetic resonance imaging (fMRI). We used cognitive and affective measures (standardized, school‐like tests of mathematics, language, and convergent and divergent creativity, as well as emotion regulation strategies and school‐related emotions) to situate the neural findings against real‐world academic and cognitive‐emotional functioning.

**FIGURE 1:**
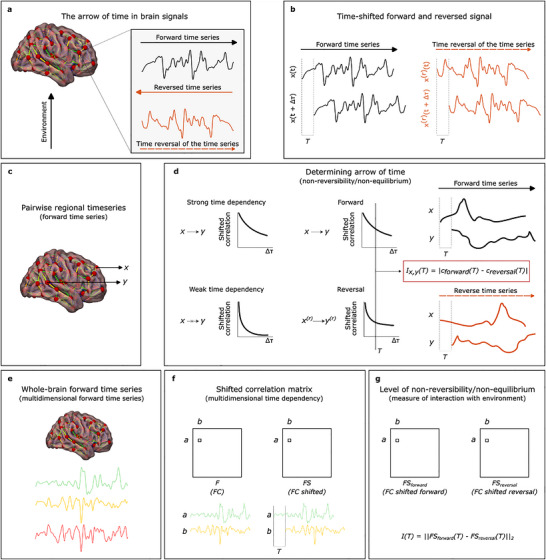
Overview of how to quantify the arrow of time to capture the inside‐out balance of intrinsic and extrinsic dynamics. Adapted under the terms of the CC‐BY license [30]. Copyright 2022, The Authors, published by Springer Nature. (a) Interaction between the brain and the environment continuously pushes the former to non‐equilibrium, and such brain activity produces entropy in a specific direction: it has a distinguishable arrow of time. (b) The arrow of time contains a precise signature of reversibility/non‐equilibrium. This is assessed through the asymmetry between the forward and the reversed time series using a time‐shifted correlation measure. This panel illustrates how a signal is reversed in time and how both the forward and reversed signals are also shifted in time. (c) This asymmetry is estimated through pairwise comparisons of signals across the whole brain. (d) The shifted correlation captures causal interactions between two‐time series. This is seen by how it decays quicker for signals with weak compared to strong time dependencies. The level of non‐reversibility is computed as the absolute quadratic difference between the time‐shifted correlations between the forward and the reversed time series. (e) We applied this framework to the whole‐brain multidimensional time series. (f) On the left of this panel, the classic NxN functional connectivity (FC) matrix of correlations between pairs of regions is illustrated. On the right, see the time‐shifted correlation matrix we used in this framework. (g) We created two time‐shifted correlation matrices for the forward and the reversed time series. The level of non‐reversibility is given precisely by the distance between these two matrices.

Our central neural measure was a valence‐free systems marker of brain dynamics: temporal asymmetry (“non‐reversibility”) of fMRI signals, estimated with the INSIDEOUT framework [30]. Non‐reversibility, a thermodynamics‐inspired metric, quantifies “arrow of time” in neural activity, reflecting a system's deviation from an equilibrium state and the complexity of its functional hierarchies, both key neural signatures of advanced cognitive functioning [31]. Large‐scale complex systems in nature (e.g., weather patterns, economy) never stay in perfect balance, but evolve in a particular direction and create disorder, or what is known as ‘entropy’. The direction of entropy production determines the arrow of time. Similarly, the brain, a complex system if there ever was one, operates optimally when it is in an active, unbalanced state, consuming the energy our body feeds it: it is far away from equilibrium [32], and gets closer to equilibrium in states of unconsciousness or reduced consciousness [33]. Furthermore, non‐reversibility of brain activity is closely linked to the strength and complexity of the functional hierarchy [31] in which the brain organizes itself to be able to perform efficient, fast computations and adapt to environmental demands [34, 35]. Thus, Deco and colleagues introduced a quantitative measure of the presence of the arrow of time in the brain by measuring the degree of non‐reversibility of neuroimaging data [30]. That is, this method measures how distinguishable the forward evolution of brain activity is from its time‐reversal by comparing the time‐shifted correlation matrices for the forward and reversed time series (see an overview of this method in Figure 1). They demonstrated that non‐reversibility in neural activity is essential for conscious awareness, as this measure was able to distinguish extremely well between wakefulness, deep sleep, and anesthesia conditions using electrocorticographical data from non‐human primates. Cumulative studies corroborate the idea that the complexity and strength of functional hierarchies are related to fast, efficient computation and adaptive behavior [34, 35]. Non‐reversibility, a proxy of the former, has been linked to greater cognitive engagement, environmental responsiveness, and the activation of endogenous, flexible processing [30, 33, 36, 37, 38, 39]. Thus, we interpret increased non‐reversibility with age as a potential marker of the maturing brain's ability to self‐organize in increasingly complex, context‐sensitive ways. Furthermore, in human fMRI data, non‐reversibility has been shown to be essential to support the cognitive demands of tasks, as it increased for seven different cognitive tasks in comparison to rest [30]. The specific mathematical formulation of the INSIDEOUT framework has also been successfully used in several other contexts: to distinguish neurodegenerative disease patients from healthy controls [40, 41], to characterize brain dynamics of patients with diseases of consciousness [37], and to distinguish different brain states in both animal and human studies [38, 42]. Thus, it is a sensitive measure of dynamic brain complexity and reflects the brain's ability to operate optimally. At rest, higher non‐reversibility indicates a richer repertoire of spontaneous, internally driven neural dynamics. In this study, we posited that it would be sensitive enough to distinguish the effects of different schooling experiences in students and help us identify which neural networks were key to these differences.

We hypothesized that educational environments aligned with developmental needs, those that gradually relax external control and promote self‐driven learning, would more effectively support the emergence of mature neural signatures of flexible, endogenous brain dynamics. Conversely, we hypothesized that environments that impose increasing external constraints might dampen the flexibility and spontaneity of endogenous brain dynamics, lowering non‐reversibility. Thus, we hypothesized that whole‐brain levels of non‐reversibility during resting state (i.e., undirected attention) would increase from lower to higher levels over age in MSC, while a reversed pattern would be observed in TSC, mirroring reciprocal shifts in the learning environments to which the students are exposed. Because the movie‐watching condition engages externally guided attention and reflects comparable screen exposure across groups, we did not expect to observe parallel age‐by‐pedagogy interactions. Instead, we hypothesized that MSC would show an advantage from a young age (in terms of more mature patterns of neural organization) during directed attention. We further anticipated that group‐related effects would localize to functional systems theoretically linked to differences in pedagogical practice: the dorsal and ventral attention networks, given the uninterrupted vs. interrupted work cycles in MSC and TSC [43]; and the frontoparietal, limbic, and default mode networks, which have been associated with cognitive flexibility and creative cognition. [44, 45, 46, 47] The dorsal attention network supports top‐down allocation of attention and goal‐directed information selection, whereas the ventral attention network includes the temporoparietal junction and ventral frontal cortex and is implicated in stimulus‐driven, bottom‐up attentional reorienting. The frontoparietal network comprises lateral prefrontal and posterior parietal regions and underlies adaptive cognitive control. The limbic network encompasses orbitofrontal, temporal pole, and subcortical structures central to affective evaluation and motivation. Finally, the default mode network includes medial prefrontal cortex, posterior cingulate cortex, and angular gyrus, supporting internally oriented cognition, self‐generated thought, and spontaneous associative processing. Differences in neural activity within these brain regions have already been observed when comparing MSC and TSC [48, 49, 50, 51], providing a theoretically relevant framework for examining potential contrasts in neural dynamics.

Finally, we conducted an exploratory analysis of sex differences between MSC and TSC on neural dynamics. Based on past work revealing sex differences in TSC compared to MSC [52], we hypothesized that male and female participants would show more substantial differences in the TSC group than in the MSC group, where more externally regulated learning environments may amplify gender‐related variation in cognitive‐emotional engagement, whereas the Montessori environment may mitigate such disparities. We did not examine sex differences in cognitive or affective outcomes because our sample size, once stratified by pedagogy and age group, did not provide sufficient statistical power for reliable interaction estimates. Given these limitations, we restricted our exploratory analyses of sex to neural measures, where effect sizes tend to be larger and where prior work suggests pedagogical context may modulate sex‐related variability.

## 2 Results

### 2.1 Demographics and Group Variables

We selected age 8.5 as a threshold. Importantly, this threshold was not chosen arbitrarily but reflects both sample‐specific and theoretical considerations. First, it is approximately the median split in our data (see Figure S1 for age histograms), therefore ensuring balanced group sizes for comparison. Second, converging evidence from developmental neuroscience indicates that this period marks a meaningful transition in large‐scale brain organization [14, 19], one that pedagogical practices are expected to mirror through increasing opportunities for self‐direction. Prior to age 8, functional brain networks undergo substantial synaptic pruning and myelination, alongside early phases of network segregation and long‐range integration, changes that scaffold the emergence of foundational executive functions, attentional control, and behavioral regulation [53]. Around age 8, children typically begin shifting from predominantly concrete learning toward more abstract reasoning, reflective thinking, and metacognitive awareness [54, 55], reflecting higher hierarchical integration of large‐scale functional networks. Finally, by age 8.5, students in Montessori and traditional systems have typically spent approximately two years within their respective environments, enough time for pedagogical context to meaningfully shape developmental patterns of neural dynamics.

MSC and TSC were comparable in age, sex, handedness ratio, and fluid intelligence (all *p* > 0.293). Parental contexts and lifestyles were similar in terms of socioeconomic status, interest in pedagogy, number of activities done with their child, parents’ perception of life as stressful, whether their child plays an instrument or practices an extracurricular activity, has access to green space, and average daily time spent watching TV (all *p* > 0.076, see Table 1).

**TABLE 1:** Demographics and group variables.

Mean (SD)	Traditional (N = 53)	Montessori (N = 43)	Missing data	Statistics	Effect size
Age	9.34 (2.27)	8.86 (2.17)	0	*t*(94) = 1.03, *p =* 0.304	0.212
Sex (girls)	54.72%	46.51%	0	χ^2^ = 0.64, *p =* 0.424	
Handedness (left)	5.66%	11.63%	0	χ^2^ = 1.11, *p =* 0.293	
Fluid intelligence	32.0 (3.86)	32.4 (4.10)	2	*t*(92) = 0.56, *p =* 0.579	0.115
Socioeconomic status	3.10 (0.60)	2.94 (0.48)	2	U = 942, *p* = 0.249	0.137
Interest for pedagogy	2.48 (0.73)	2.67 (0.53)	2	U = 975, *p* = 0.290	0.108
Number of activities done with their child	8.22 (2.36)	9.07 (1.81)	3	U = 862, *p* = 0.102	0.195
Perception of life as stressful (‘yes’)	44.90%	30.00%	7	χ^2^ = 2.07, *p* = 0.150	
Instrument (‘yes’)	44.23%	40.48%	2	χ^2^ = 0.13, *p* = 0.714	
Extra‐scholar activity (‘yes’)	92.31%	95.24%	2	χ^2^ = 0.33, *p* = 0.563	
Access to green space (‘yes’)	92.31%	100%	5	χ^2^ = 3.14, *p* = 0.076	
Daily time spent watching TV (‘<1 h/day’)	80.95%	72.55%	3	χ^2^ = 6.37, *p* = 0.095	

### 2.2 Differences Between MSC and TSC in Cognitive Variables by Age Group

A multivariate ANCOVA (MANCOVA) conducted on the six cognitive and affective outcome variables revealed a significant multivariate main effect of pedagogy (Pillai's trace = 0.303, F(6, 50) = 3.63, *p* = 0.0046), indicating that the joint profile of academic, creative, and affective outcomes differed reliably between students in traditional and Montessori classrooms after adjustment for socio‐economic status (SES) and fluid intelligence. SES also exhibited a significant multivariate effect (Pillai's trace = 0.281, F(6, 50) = 3.25, *p* = 0.0089), and fluid intelligence contributed significantly to the multivariate outcome pattern (Pillai's trace = 0.220, F(6, 50) = 2.36, *p* = 0.0441). In contrast, neither age group nor the pedagogy × age group interaction yielded significant multivariate effects (age group: Pillai's trace = 0.180, F(6, 50) = 1.83, *p* = 0.112; interaction: Pillai's trace = 0.098, F(6, 50) = 0.90, *p* = 0.50), suggesting that the pedagogical differences did not vary measurably between the younger and older students in this sample, see Table 2.

**TABLE 2:** Summary of Type III ANCOVA results for cognitive and affective outcomes. Pedagogy (TSC vs. MSC) and Age group (<8.5 vs. ≥8.5 years) entered as fixed factors; SES and fluid intelligence as covariates.

Outcome	Domain	Pedagogy *p* ^a^	Age group *p* ^a^	Interaction *p* ^a^
Math	Academic	0.039	0.347	0.510
Language	Academic	<0.001	0.943	0.776
Divergent thinking	Creativity	0.002	0.553	0.211
Convergent thinking	Creativity	<0.001	0.639	0.614
Emotion regulation (ERQ)	Affective	0.378	0.662	0.904
Epistemic emotions	Affective	0.010	0.196	0.152

^a^ p‐values correspond to Type III tests from ANCOVAs of the form Y ∼ SES + fluid_intelligence + Pedagogy × Age_group, with sum‐to‐zero contrasts.

**FIGURE 2:**
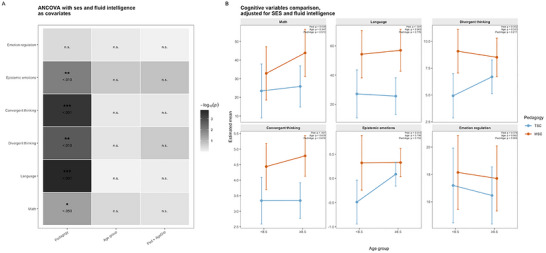
Pedagogy‐related differences across cognitive and affective outcomes. A. Heatmap of p‐values from the ANCOVA models testing effects of pedagogy (TSC, *n* = 53 vs. MSC, *n* = 43), age group (<8.5 vs. ≥8.5 years), and their interaction on six outcome variables (math, language, divergent thinking, convergent thinking, emotion regulation, epistemic emotions), while adjusting for SES and fluid intelligence. Color intensity represents the –log_10_(p) value for each term‐outcome variable combination. Significance is displayed using both symbols (^*^ *p* ≤ 0.05, ^**^ *p* ≤ 0.01, ^***^ *p* ≤ 0.001) and numerical thresholds (e.g., “<.010”). Effects occur for academic and creative cognition, as well as emotion experienced at school, with no reliable interactions involving age groups. B. Estimated marginal means (EMMs) ± 95% confidence intervals for each outcome, plotted separately for TSC (*n* = 53) and MSC (*n* = 43) and stratified by age group. Consistent with Panel A, MSC students show higher adjusted performance in math, language, and both divergent and convergent thinking, as well as more adaptive epistemic emotions, while emotion regulation does not differ significantly between groups.

Follow‐up univariate ANCOVAs clarified how the multivariate pedagogy effect was expressed across individual outcomes (Figure 2). For math, pedagogy exerted a significant main effect (*p* = 0.039). Adjusted mean math scores were higher in MSC classrooms (EMM = 38.4, SE = 4.75) than in TSC (EMM = 24.7, SE = 4.38), yielding an estimated mean difference of −13.7 points (TSC – MSC = −13.7, SE = 6.55, *p* = 0.039). The effect was even stronger for language (*p* = 1.64 × 10^−^^4^). Students in MSC scored nearly 30 points above those in TSC after covariate adjustment (TSC: EMM = 26.4, SE = 4.96; MSC: EMM = 55.6, SE = 5.38; contrast = −29.2, SE = 7.42, *p* = 0.0002). For both academic domains, neither age group nor the pedagogy × age group interaction was significant (all *p* > 0.34), indicating consistent pedagogical advantages across age groups.

For divergent thinking, the main effect of pedagogy was significant (*p* = 0.0019). MSC students exhibited higher scores (EMM = 8.80, SE = 0.68) than TSC (EMM = 5.81, SE = 0.62), an estimated difference of −2.99 points (SE = 0.93, *p* = 0.0019). A similar pattern was observed for convergent thinking (*p* = 0.00034). Children in Montessori schools outperformed TSC (MSC: EMM = 4.61, SE = 0.25; TSC: EMM = 3.34, SE = 0.23; difference = −1.27, SE = 0.34, *p* = 0.0003). Age group and the interaction did not reach significance (all *p* > 0.21).

For the emotion regulation measure (reappraisal), there was no evidence for a pedagogical effect (*p* = 0.378), nor for age group or the interaction (*p* ≥ 0.66). General emotion regulation abilities, therefore, appeared comparable across pedagogical environments. In contrast, epistemic emotions differed significantly between pedagogies (*p* = 0.0099). MSC students displayed more adaptive epistemic emotional profiles (EMM = 0.324, SE = 0.156) than TSC students (EMM = −0.201, SE = 0.122), corresponding to an adjusted mean difference of −0.525 (SE = 0.196, *p* = 0.0099). Age group and the interaction were not significant (*p* = 0.196 and *p* = 0.152, respectively).

### 2.3 Differences Between MSC and TSC in Non‐Reversibility by Age Group

**FIGURE 3:**
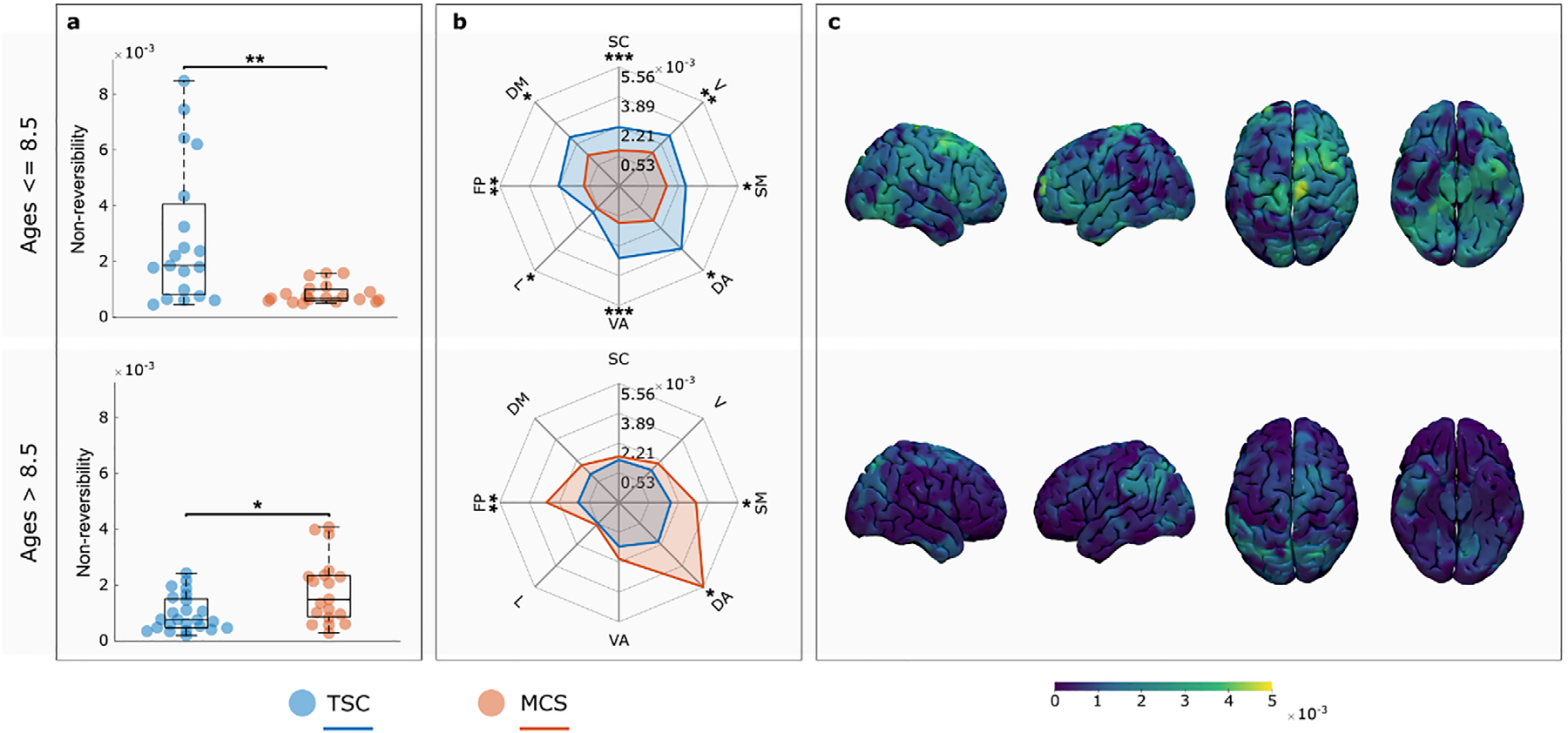
Traditionally schooled children (TSC) and Montessori schooled children (MSC) show significant differences in non‐reversibility levels of resting state activity depending on age group. (a) For children younger than 8.5 years old (top row; TSC, *n* = 19; MSC, *n* = 19), we found higher global levels of non‐reversibility for TSC than for MSC (*p*  ≤  0.01, SSMD = 0.79). In contrast, for students older than 8.5 years old (bottom row; TSC, *n* = 24; MSC, *n* = 19), we found lower global levels of non‐reversibility for TSC than for MSC (*p*  ≤ 0.05, SSMD = 0.61). (b) We found similar changes in local brain networks: Subcortical (SC), Visual (V), Sensorimotor (SM), Dorsal Attention (DA), Ventral Attention (VA), Limbic (L), Frontoparietal (FP), and Default Mode (DM). For the younger group (top row), the most significant changes were found in VA and SC networks (*p*  ≤ 0.001). For students older than 8.5 years old (bottom row), the most significant changes were found in the FP network (*p*  ≤  0.01). Radar plots show each group's median values. (c) We rendered the regional changes in terms of absolute difference between average node values of each group (MSC vs. TSC) onto a 3D brain model. Statistical significance was assessed with a two‐sided Monte Carlo permutation test on the medians. * represents *p*  ≤ 0.05, ** represents *p*  ≤  0.01, and *** represents *p*  ≤ 0.001.

In the resting state condition (i.e., no directed attention), TSC younger than 8.5 years old exhibited higher levels of non‐reversibility than same‐age MSC. All functional systems led to this whole‐brain difference. This effect reversed for older students. This is illustrated in Figure 3a, with boxplots showing global non‐reversibility. The attentional, frontoparietal, and sensorimotor networks led to these differences (see Figure 3b, where the non‐reversibility levels for each functional network are illustrated as radar plots). The distribution of average non‐reversibility values of each node was most different between the groups for younger children (Figure 3c).

**FIGURE 4:**
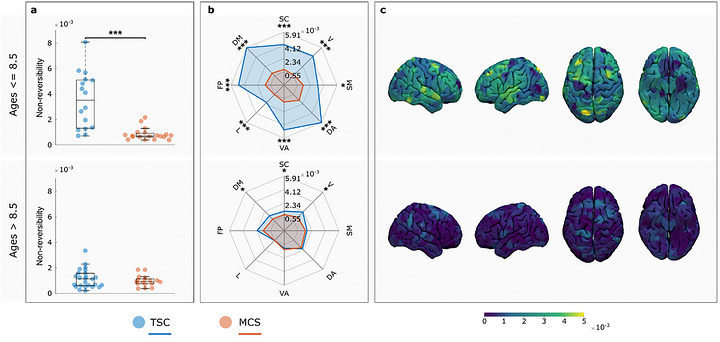
Traditionally schooled children (TSC) and Montessori schooled children (MSC) show significant differences in non‐reversibility levels of brain activity during movie‐watching, depending on age group. (a) Similar to the results for resting state activity, children younger than 8.5 years old (top row; TSC, *n* = 16; MSC, *n* = 19) showed higher global levels of non‐reversibility for TSC than for MSC (*p*  ≤ 0.001, SSMD = 1.18). In contrast, there were no significant changes (SSMD = 0.2) at the global level between TSC and MSC older than 8.5 years old (bottom row; TSC, *n* = 25; MSC, *n* = 17). (b) By networks: Subcortical (SC), Visual (V), Sensorimotor (SM), Dorsal Attention (DA), Ventral Attention (VA), Limbic (L), Frontoparietal (FP), and Default Mode (DM). For children younger than 8.5 years old (top row), again, significant changes in non‐reversibility levels were found for all brain networks. Much less so for students older than 8.5 years old (bottom row); however, the difference was still significant in the DM, SC, and V networks (*p*  <  0.05). Radar plots show each group's median values. (c) Absolute difference between median node values of each group rendered onto a 3D brain model. Statistical significance was assessed with a two‐sided Monte Carlo permutation test on the medians. * represents *p*  ≤ 0.05, ** represents *p*  ≤  0.01, and *** represents *p*  ≤ 0.001.

In the movie‐watching condition (i.e., directed attention), TSC younger than 8.5 years of age exhibited higher levels of non‐reversibility than their Montessori‐schooled peers in all functional systems, as we found for the rest condition. This leveled as students got older; no global difference was seen between TSC and MSC among students aged 8.5 years or older (Figure 4). However, default mode, subcortical, and visual networks still showed higher levels of non‐reversibility in TSC than MSC.

### 2.4 Differences Between MSC and TSC in Non‐Reversibility by Sex

**FIGURE 5:**
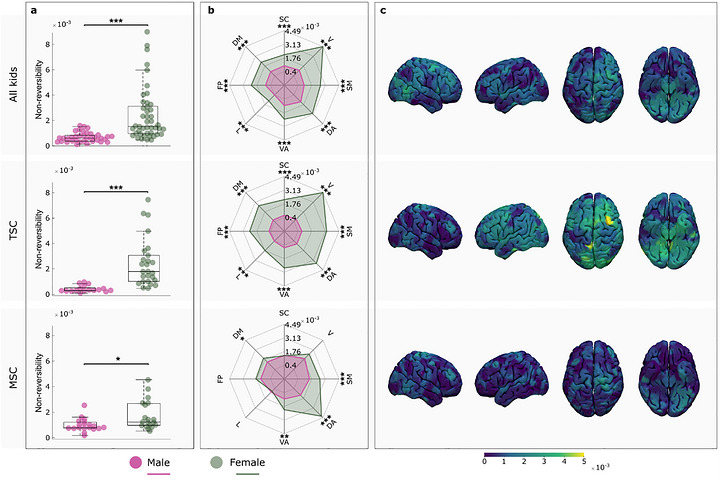
Sex‐differences in resting state activity in traditionally schooled children (TSC) and Montessori schooled children (MSC). (a) Non‐reversibility at the global level was significantly higher for female students compared to male students, when grouping for all students (top row; Male, *n* = 36; Female, *n* = 45; *p*  ≤ 0.001; SSMD = 0.82), for TSC (middle row; Male, *n* = 17; Female, *n* = 26; *p*  ≤ 0.001; SSMD = 1.05) and for MSC (bottom row; Male, *n* = 19; Female, *n* = 19; *p*  ≤ 0.05; SSMD = 0.62). (b) Significant changes were also found in local brain networks: Subcortical (SC), Visual (V), Sensorimotor (SM), Dorsal Attention (DA), Ventral Attention (VA), Limbic (L), Frontoparietal (FP), and Default Mode (DM). Radar plots show each group's median values. (c) The regional changes in absolute differences between median node values for each group were rendered onto a 3D brain model. Statistical significance was assessed with a two‐sided Monte Carlo permutation test on the medians. * represents *p*  ≤ 0.05, ** represents *p*  ≤  0.01, and *** represents *p*  ≤ 0.001.

Another potentially critical factor in students' neurodevelopment is sex: there are known sex differences in neurodevelopment and epigenetics [56]. Therefore, as an exploratory analysis, we compared non‐reversibility levels by sex at rest and movie conditions. We found that females had higher non‐reversibility levels across the whole brain (see Figure 5a). Sex differences were more marked among TSC than MSC. We computed the SSMD between the global non‐reversibility values of students of each sex as a measure of effect size: SSMD = 0.62 in the MSC group and SSMD = 1.05 in the TSC group. This is also clearly seen in Figure 5b, with the non‐reversibility levels illustrated as radar plots. You can also see here that the dorsal attention network leads to the difference between sexes in the MSC group.

**FIGURE 6:**
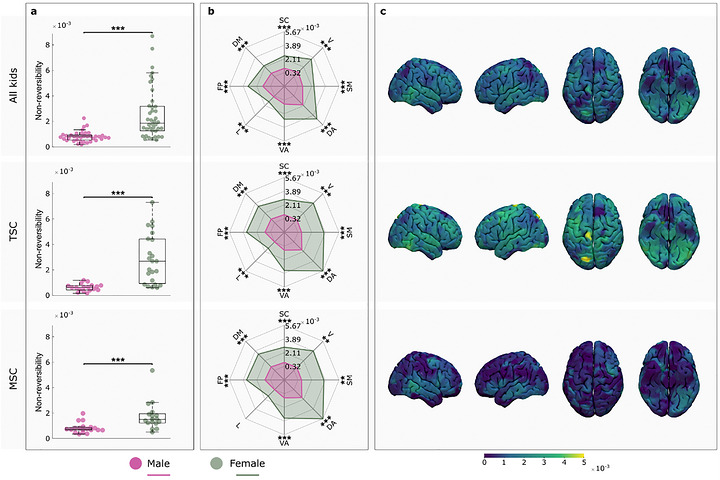
Sex‐differences in non‐reversibility levels during movie watching when comparing traditionally schooled children (TSC) and Montessori schooled children (MSC). a) Similar to the results for resting state activity, we found that global levels non‐reversibility for movie‐watching were significantly higher for female students compared to male students, when grouping for all children (top row; Male, *n* = 37; Female, *n* = 40; *p*  ≤ 0.001; SSMD = 0.86), for TSC (middle row; Male, *n* = 18; Female, *n* = 23; *p*  ≤ 0.001; SSMD = 1.12) and for MSC (bottom row; Male, *n* = 19; Female, *n* = 17; *p*  ≤ 0.001; SSMD = 0.79). (b) Again, significant changes were also found in local brain networks: Subcortical (SC), Visual (V), Sensorimotor (SM), Dorsal Attention (DA), Ventral Attention (VA), Limbic (L), Frontoparietal (FP), and Default Mode (DM). Radar plots show each group's median values. (c) The regional changes in absolute differences between median node values for each group were rendered onto a 3D brain model. Statistical significance was assessed with a two‐sided Monte Carlo permutation test on the medians. * represents *p*  ≤ 0.05, ** represents *p*  ≤  0.01, and *** represents *p*  ≤ 0.001.

Analogously, in the movie condition, we also observed that females showed higher levels of non‐reversibility at the global level (Figure 6a). Again, this was more pronounced within the TSC group (see Figure 6b,c). The effect size in the comparison between the global non‐reversibility values of the students of each sex was SSMD = 0.79 in the Montessori group and SSMD = 1.12 in the traditional group.

## 3 Conclusion

This study contrasted two widely used schooling trajectories: the traditional model, which tends to become increasingly adult‐directed with age, and Montessori, which gradually expands learning autonomy within a stable classroom structure, to ask whether the pedagogical approach moderates large‐scale brain dynamics and cognitive performance. Using an a priori anchor at 8.5 years, a developmentally meaningful point in functional network organization reflecting higher self‐monitoring abilities, and when school environments should provide more degrees of liberty for self‐direction, we observed opposing age trajectories in temporal asymmetry: non‐reversibility values increased with age in MSCs and decreased with age in TSCs, with the largest effects in attention, frontoparietal, and sensorimotor networks. We reported that SSMD, alongside p‐values, provides an indication of effect sizes. SSMD (which is based on the standard deviation of the difference between groups rather than a pooled variance) is a measure of effect size somewhat analogous to Cohen's d. We chose SSMD because it provides a more conservative estimate of group separation in the presence of variance heterogeneity, and because in the context of our contrast‐based feature selection approach, Cohen's d may yield inflated values for effect size, potentially misleading readers accustomed to conventional benchmarks. Across conditions, effects ranged from small (SSMD ≈ 0.2) to large (SSMD > 1.0). These values reflect separation after feature selection of the most discriminative node pairs, so they should not be directly compared to other field conventions, but as quantifying the strength of group differentiation within our high‐dimensional framework. In this context, we interpret moderate‐to‐large SSMD values as reliable differences in the temporal asymmetry structure of brain activity, while small values (e.g., in older children during movie‐watching) suggest convergence between groups. Behaviorally, the MSCs scored higher in language and mathematical skills, in divergent and convergent creativity thinking, as well as positive emotions experienced in relation to learning (i.e., epistemic emotion).

MSCs displayed a distinct increase in the non‐reversibility of their brain dynamics with age during the resting state condition (the condition of unconstrained cognition). Their TSC peers showed the reverse pattern (Figure 3). Non‐reversibility in brain dynamics, which reflects temporal asymmetry in neural signals, has been linked to conscious awareness, cognitive flexibility, and hierarchical neural processing [30, 36, 39, 57, 58]. To our knowledge, this framework has not previously been used to probe differences associated with schooling context. Indeed, we know of no other studies looking at differences in brain dynamics across different schooling contexts in a non‐causal way. The functional meaning of neural non‐reversibility across settings remains to be fully established; nonetheless, it is a sensitive measure reflecting the strength of functional hierarchies and computational complexity in the brain [31]. In this context, the developmental pattern we observed, with higher non‐reversibility in TSC students at younger ages and a steady age‐related increase in MSC students that ultimately surpassed TSC levels, suggests that Montessori education may support a late‐emerging amplification of endogenous neural dynamics. The MSC trajectory raises the possibility of continued increases beyond our age range, consistent with a “blooming” developmental profile in which hierarchical neural organization strengthens progressively across childhood and adolescence. Although Montessori education is often associated with early childhood (3–6 years) [59], Montessori theory proposes increasing autonomy after age six as part of its “planes” of development [60, 61]. The increasing non‐reversibility observed with age in Montessori students may therefore reflect this progressive shift toward more self‐directed and collaborative learning.

This pattern motivates a broader theoretical consideration: a slower early rise in non‐reversibility, followed by accelerated growth, may reflect a more adaptive maturation of large‐scale functional hierarchies. Early TSC elevations could signal premature stabilization or externally structured neural engagement, whereas the gradual MSC increase aligns with the idea that hierarchical integration benefits from extended periods of exploratory, self‐directed learning once foundational regulatory capacities are in place. From a neurodevelopmental standpoint, large‐scale dynamics begin relatively unstructured and consolidate through experience‐dependent specialization. A pedagogy that moves from clear scaffolding to increasing autonomy may therefore better parallel the natural consolidation of functional hierarchies. In this view, a slower early rise in non‐reversibility is not a deficit but may preserve degrees of freedom for later, more complex neural integration, consistent with the superior academic, creative, and socio‐emotional outcomes observed in MSC students at all ages.

At the network level, group differences were most pronounced in the sensorimotor, dorsal attention, and frontoparietal systems. These networks support action‐based learning and attentional‐executive control, processes also implicated in creativity and language. Beyond overt motor control, the sensorimotor network contributes to the cognitive mapping of action plans and skills [62, 63] and engages in aspects of semantic processing [64], capacities that are frequently exercised in hands‐on, peer‐rich settings [65, 66]. These observations are consistent with the differences observed in creativity and language skills. The dorsal attention network underpins top‐down selective attention and dynamic reallocation of attentional resources [67, 68]. The frontoparietal network functions as a flexible control hub for executive functions, integrating new with existing information and for flexibly adjusting strategies across changing task demands [69, 70, 71]. The interplay among these networks supports flexible adjustment of learning strategies as information and task demands change.

These findings are consistent with (though, of course, they do not prove) Montessori contexts scaffolding durable, adaptable cognitive frameworks that could partly account for the observed advantages on language and creativity tests in our sample. This interpretation aligns with prior comparisons of MSC and TSC, reporting superior academic performance [72], self‐direction and independent learning [73], and socioemotional skills among MSC [74, 75], with lasting effects into adolescence and adulthood [76]. Conversely, traditional approaches that emphasize efficiency on well‐specified tasks may cultivate proficiency under tight external constraints, but as argued elsewhere, [77] may offer fewer opportunities to practice broadly transferable skills for novel, ill‐defined problems. In line with this view, several studies have documented lower creative thinking abilities in older TSC students, with the reverse found for MSC [72, 78, 79]. However, longitudinal studies are needed to go beyond inferences.

Recent research on adults demonstrates that resting state shows higher levels of non‐reversibility and increased hierarchical organization than when watching a naturalistic movie [36]. This suggests that introspection or mind wandering can be solid dynamics drivers, pushing the brain toward non‐equilibrium [36]. Lower reversibility was consistently observed in the MSC groups in the movie‐watching condition (i.e., directed attention). However, the TSC group differed at a younger age in all networks, and in the subcortical, visual, and default mode networks for older students (Figure 4), even though screen habits and time spent watching TV do not differ between groups. The default mode network, involved in narrative comprehension and emotional resonance, helps viewers integrate on‐screen events with personal memories and emotions, enhancing engagement [80]. Subcortical regions such as the amygdala and hippocampus are crucial for emotional processing and memory formation, influencing emotional responses and the retention of emotionally charged scenes. [81] The visual network handles the processing of visual stimuli, from basic features to complex scene compositions, crucial for understanding the visual narrative [82]. The interaction among these networks underpins a cohesive viewing experience, where the integration of visual perception, emotional content, and narrative comprehension enriches the movie's overall impact, supporting both personal relevance and emotional depth. Taken together, these patterns raise the possibility that MSCs may rely more on internally guided, equilibrium‐leaning processing during directed tasks, whereas TSCs, especially younger ones, engage perceptual, affective, and narrative‐integration networks more asymmetrically, potentially reflecting schooling‐shaped differences in how external input is interpreted and emotionally encoded.

Importantly, resting‐state condition captures unconstrained internal cognition, including processes such as cogitation, mind‐wandering, meaning‐making, planning, self‐reflection, and spontaneous associative thought, which may vary across participants depending on engagement, fatigue, or emotional state. Thus, resting state and movie watching probe complementary regimes of cognition: resting state reflects internally‐generated neural dynamics, whereas movie watching captures externally‐guided yet interpretive engagement with the environment. These regimes may relate differently to the behavioral outcomes examined in the study, with internally‐generated dynamics supporting creative and flexible cognition and externally‐guided engagement involving attentional processes relevant to structured learning contexts.

Finally, we report that females consistently exhibited higher levels of non‐reversibility than males, with that being much more pronounced among TSC than among MSC (Figures 5 and 6). This analysis is exploratory and not causal. Several non‐exclusive interpretations are plausible. One is that differences between males and females in baseline neural dynamics partly reflect biological factors, including differences in maturation rates and hormonal influences that have been linked to sex‐specific patterns of functional organization [83, 84], and the Montessori environment decreases those differences, perhaps by offering a more autonomy‐supportive framework [56, 85]. Another possibility is that traditional school context amplifies such differences, where more rigid structure, externally imposed expectations, and competitive performance norms may interact with gendered socialization and classroom management practices: for example, boys’ higher motor activity and more overt exploratory behavior are more likely to be labeled as disruptive, redirected, or sanctioned, whereas girls' relatively compliant and verbally mediated behaviors are more often positively reinforced [85, 86]. Reliance on external regulation, via adult‐led instruction and standardized assessments, may constrain this flexibility, mirroring progression toward rule‐based moral reasoning highlighted in Gilligan's critique of conventional moral development models [87]. Over time, such asymmetries could constrain boys’ opportunities to engage in flexible, self‐initiated problem solving and emotional expression, effectively narrowing the repertoire of neural states they routinely occupy.

Conversely, Montessori education's emphasis on self‐directed and flexible learning may mitigate these differences. This pedagogy systematically requires all children, regardless of sex, to generate their own strategies, monitor their performance, and adjust their behavior without constant external evaluation, thereby reducing opportunities for adult gendered biases to shape children's learning behaviors. These results are in line with sex differences observed in another recent study in the field of neuroscience and Montessori education, where consistently, TSC male and female students differ in brain morphometry markers of stress, while not MSCs [52]. This is an important topic, and a better understanding of where these differences stem from should help in tailoring educational practices to support optimal cognitive and neural development for all learners in a truly inclusive way. We call for more work investigating how pedagogical practices at critical ages may help empower females’ and males' development beyond diversity, but as a foundation for complementarity and mutual empowerment in learning environments.

Overall, these findings support the notion that pedagogy, beyond curricular content, plays a critical role in shaping the organization of neural dynamics in measurable ways. While we lack a definitive explanation for the group differences observed among younger learners – which may stem from pre‐existing individual differences, school influences that may early nurture less reversible neural dynamics, or selection biases – a pedagogical strategy that aligns with neurodevelopmental stages (i.e., Montessori) appears to foster neural organization that supports adaptability and conscious cognitive engagement across development and may reduce disparities among the sexes. These findings are preliminary but foundational. Future studies could explore more homogenous educational systems that emphasize either less‐structured environments, such as the pedagogy of discovery common in Finnish educational culture, or highly structured environments prevalent in many Asian cultures, consistently throughout all stages of schooling.

Some limitations must be acknowledged. We have demonstrated that there is a distinct effect from different schooling systems on the functional architecture of the brain, but the underpinning mechanisms and the extent to which observed group differences in neural dynamics explain group differences in cognition remain speculative and an avenue for future research. While we did our best to counter the effect of selection bias inherent to Montessori schools being the only private schools in Switzerland, we cannot exclude that families enrolling their children in alternative schooling systems might have traits that partially confound the findings observed. Ideally, similar research should be conducted in countries where Montessori, or similar pedagogies, are implemented in public schools. We also recommend following the same students over time to better understand causal relations between characteristics of schooling and brain development, and how intra‐individual hierarchical neural dynamic organization changes. Finally, to properly study and characterize the impact of structured vs. less‐structured learning environments at different ages would require large‐scale studies enrolling learners from multiple schooling backgrounds. Is it better for a young mind to be first in a framed setting and learn actions in a more guided way and later build and explore more freely, or the other way round? Which approach leads to more optimal neural development and joy in learning, studying, and working collaboratively as a team? These are important questions to address to respond to societal challenges. Scientific approaches will need to align with these research questions.

This study is the first to demonstrate that across development, different pedagogical models are differentially mirrored in the hierarchical neural dynamic organization of learners. Current findings suggest that, rather than engaging in dualistic debates over highly structured vs. less‐structured learning environments, the most effective approach might be a unified framework that aligns with the child's biological development. This approach highlights the importance of tailoring educational experiences to individual developmental stages [55, 88], which we propose can lead to more effective and supportive learning outcomes, as observed in MSC.

## 4 Materials and Methods

### 4.1 Participants

A large study investigating the effect of school experience on learners’ neurodevelopment has been ongoing since 2018 [89]. As part of this initiative, participants were recruited from over 15 collaborating schools across the francophone region of Switzerland and invited to take part in laboratory sessions at the Lausanne University Hospital (CHUV‐UNIL). All schools were standard co‐educational institutions. Eligible participants were between the ages of 4 and 18 years, had either a Montessori or a traditional schooling background, and had no history of neurological or learning disorders as reported by their parents. When the current analyses were performed, data from 111 students were available. Data from students who transitioned from one schooling system to another were removed (i.e., no ‘clear’ schooling background). Additionally, 10 participants were removed due to motion artifacts during scanning (*n* = 5) or MRI incompatibility related to dental braces (*n* = 5). The final sample for the current study consisted of 96 learners (age range: 4.6–15.2 years; mean age: 9.12 ± 2.22 years; 49 girls [51%]). Of these, 43 attended Montessori schools (mean age: 8.86 ± 2.17 years; 20 girls) and 53 attended traditional schools (mean age: 9.34 ± 2.27 years; 29 girls).

All participants provided oral and written consent. This was obtained from a legal guardian for participants younger than 14 years. The study was approved by the Commission cantonale d'éthique de la recherche sur l'être humain du canton de Vaud (CER‐VD), Switzerland (approval number: 2018‐00244).

### 4.2 Demographics and Group Variables

In Switzerland, Montessori education is offered exclusively in private institutions. To account for potential selection bias associated with the schooling system, we collected demographic and background variables to ensure comparability between MSC and TSC. We obtained data on family environment, including parents’ socio‐economic background, their interest in pedagogy, the number of activities they engage with their children at home, as well as their perception of life as stressful, whether their child plays an instrument, and does an extracurricular activities (e.g., sport), has access to a green space at home, as well as the time their child spends watching TV daily (see operationalizations below). We also assessed whether the groups were matched in terms of age, sex ratio, handedness, and general cognitive abilities (i.e., fluid intelligence).

Data from the child were collected in the lab before or after the scanning session. Depending on whether the parents were present at the lab (e.g., older participants came without their parents), parental data were collected via questionnaires administered either online or in paper format (see Figures S2–S8).

The child's fluid intelligence was measured via a black‐and‐white, paper‐based version of the Raven's Progressive Matrices test (PM‐47) [90]. The assessment included 36 items divided into three sets of 12 matrices. Each matrix featured a missing element, and students were asked to choose the correct option (from six or eight possible answers) to complete the pattern. The test lasted approximately 15 min. A total score ranging from 0 to 36 was calculated by summing up the number of correct responses.

SES: Parents provided information about their educational background and professional status [91]. Responses from each parent were combined and averaged in cases of shared parental authority. For single‐parent households, the score from the sole guardian was used. The SES score had a maximum value of 4.

Interest in pedagogy: To assess their engagement with educational topics, parents responded to three questions concerning their interest in pedagogy and child development (e.g., “Do you read books about child development?”, see Figures S2–S6). The total score, with a maximum of 3, was computed by summing responses across the items.

Number of activities done with their child: Parents were asked about the frequency of specific activities shared with their child (e.g., visiting a museum, reading a book together, watching a movie together, cooking together). Low to high frequency was scored from 0 to 1 for each of the 11 questions and summed for the final score.

Categorical questions (i.e., ‘yes, ‘no’) about parents’ perception of life as stressful and whether their child plays an instrument or is involved in an extracurricular activity and has access to a green space (i.e., garden) were scored as 0 or 1.

Estimation of daily time spent watching TV: parents were asked to estimate the hours their child usually watched TV (0 h, less than 1 h, 1–2 h, or more than 2 h).

### 4.3 Cognitive and Affective Variables

Cognitive measures were administered, and those selected for this study target core domains relevant to school learning and self‐regulation: academic achievement (literacy and numeracy), creative production (divergent and convergent thinking). Affective variables selected were epistemic emotions and emotion regulation.

Academic outcome measures. Standardized literacy (“language”) and numeracy (“math”) tasks were administered. For children ≤6 years: (i) oral comprehension: 27 sentences with 4‐picture multiple choice; correct responses summed (max = 25) [92]; (ii) early reading competence: phonological awareness (deleting initial syllable, 10 items; or initial phoneme, 24 items; max = 34) and timed decoding of 30 pseudowords in 1 min (max = 30), accuracy across reading subtasks was summed and expressed as percent correct [93]; (iii) verbal problems: 10 orally presented word problems, scored for accuracy and reported as percent correct (max = 10) [94]. For learners >6 years: language competence: comprehension, grammar, and spelling after silent reading of a narrative; performance reported as percent correct; mathematics: mixed arithmetic, logic, and geometry items; performance reported as percent correct [95].

Creativity measures. Divergent and convergent production were assessed with the Evaluation du Potentiel Créatif (EPoC) standardized abstract‐drawing tasks [96]. To assess divergent thinking, participants were given 10 min to generate as many distinct drawings as possible from a single imposed abstract form; score = number of valid productions integrating the stimulus into a novel concept. To assess convergent thinking, participants were given 15 min to select ≥3 shapes from 8 and integrate them into a single drawing; three independent, group‐blind judges rated the products on a 7‐point scale (1–7) for originality and narrative coherence; score expressed as a percentage of the scale maximum.

Epistemic emotions measure. Epistemic emotions are feelings that arise during learning or thinking. We assessed them with the Epistemic Emotions Questionnaire [97], which asks learners to rate how often they experience curiosity, enjoyment, confusion, frustration, boredom, and anxiety during learning on a 5‐point Likert scale (1 = never to 5 = very often). For interpretability, negatively valenced subscales (confusion, frustration, boredom, anxiety) were reverse‐scored so that higher values uniformly indicate more adaptive epistemic engagement. A composite score was computed as the sum of all sub‐scales.

Emotion regulation measure. We assessed emotion regulation abilities with the child‐appropriate French adaptation of the Emotion Regulation Questionnaire (ERQ) [98]. The ERQ is comprised of 10 items rated on a 7‐point scale (1 = never to 7 = very frequently) and yields two subscales (cognitive reappraisal and expressive suppression). Subscale scores of cognitive reappraisals were computed as the mean of respective items, with higher values indicating higher abilities to re‐evaluate emotions, taking into account context and enlarged perspectives to regulate them.

### 4.4 fMRI Data Acquisition

Magnetic Imaging Resonance (MRI) acquisition was performed at the Lemanic Biomedical Imaging Center (CIBM) of the University Hospital of Lausanne (UNIL‐CHUV) using a 3T PrismaFit MR scanner equipped with a 64‐channel head‐coil (Siemens Healthineers, VE11E Software version, Erlangen, Germany). A description of the fMRI data acquisition procedure is also available in [89].

Structural imaging was acquired using a Magnetization Prepared—RApid Gradient Echo (MPRAGE) 3‐d high‐resolution isotropic T1‐weighted sequence (TR = 2000 ms; TE = 2.47 ms; 208 slices; voxel size = 1 × 1 × 1 mm; flip angle = 8°). These images were used for surface reconstruction.

Functional Magnetic Resonance Imaging (fMRI) data were acquired using a gradient‐echo echo‐planar imaging (EPI) sequence combined with Simultaneous Multi‐Slice (SMS) acceleration to enhance temporal resolution. Functional acquisition covered the whole brain (Gradient‐echo (GRE) 2.2 × 2.2 × 3 mm, Repetition Time (TR) = 500 ms; Echo Time (TE) = 33 ms; 48 axial slices; slice thickness = 2.6 mm; 10% gap between slices; flip angle = 47°; Field of View (FOV) = 224 mm; SMS acceleration factor = 8). Each run lasted 6 min and yielded 720 volumes. Two acquisition sessions were done for most participants, randomly ordered (i.e., when running out of time, one of the two sessions could not be done, see below for details). In one of the acquisition sessions, we acquired fMRI data in a movie‐watching condition, where the child was asked to watch a tailor‐made ‘Tom & Jerry’ 6‐min movie displayed on the screen (i.e., directed attention). We selected old ‘Tom & Jerry’ excerpts to ensure a pleasurable yet unfamiliar experience for the participants. All participants were exposed to the same film. In the other acquisition session, we recorded activity during ‘resting state’, where the child was asked to keep the eyes open and stare at a cross displayed at the center of the screen (i.e., no directed attention). Each participant was given earplugs to avoid discomfort caused by scanning noise and had pads placed around their ears to limit movement.

For the movie‐watching condition analysis, usable data were obtained from 77 learners aged between 4.6 and 12.8 years (mean age = 8.99 years, SD = 1.99; 40 girls). Among these participants, 37 were enrolled in Montessori programs (ages 4.6–12.7; mean = 8.75 years, SD = 1.93; 17 girls), and 40 attended traditional schools (ages 5.2–12.8; mean = 9.22 years, SD = 2.04; 23 girls).

For the resting state condition, data from 81 learners (ages 4.6–15.2; mean = 9.13 years, SD = 2.28; 45 girls) were analyzed. This group included 38 learners from Montessori schools (ages 4.6–14.6; mean = 8.84 years, SD = 2.24; 19 girls) and 43 from traditional schools (ages 5.2–15.2; mean = 9.39 years, SD = 2.31; 26 girls).

### 4.5 Preprocessing fMRI Data

All imaging data were preprocessed with fMRIPrep 20.2.1 [99] based on Nipype 1.5.1 [100]. The pipeline included spatial normalization of anatomical scans to the MNI152NLin2009cAsym template, brain tissue segmentation, and cortical surface reconstruction. Simultaneously, blood‐oxygen‐level‐dependent (BOLD) contrast time series from functional scans were processed to create brain masks and estimate motion parameters. Functional scans were then co‐registered to the anatomical data to ensure accurate spatial alignment. We used the Independent Component Analysis—Automatic Removal Of Motion Artifacts (ICA‐AROMA) pipeline as a retrospective resting state fMRI denoising method [101]. Time series were extracted from the preprocessed fMRI data using the spatiotemporal connectome pipeline [102];.s://github.com/agriffa/STConn), based on the Lausanne2018 atlas (506 brain regions).

To assess potential motion confounds, group differences in mean framewise displacement and the derivative of root mean square variance (DVARS) across successive volumes were examined using ANOVA. No significant effects of pedagogy, gender, or pedagogy‐related interactions were observed in either condition (resting state and movie watching, all *p* >0.08), although DVARS showed a main effect of age group (<8.5 vs. >8.5) consistent with known developmental differences in motion (see Figure S9).

### 4.6 INSIDEOUT Framework

Differences in experience and the extent to which we can perform tasks efficiently are reflected by neural dynamic activity [103, 104, 105, 106], even at rest [107]. There are a variety of ongoing research efforts to develop measures of dynamical complexity able to capture signatures of brain states and/or differential characteristics of brain dynamics [103, 104, 108, 109]. Here, we used the INSIDEOUT framework [30], a promising analysis method motivated by the recently proposed ‘thermodynamics of mind’ theory [31]. This method leverages the thermodynamics‐based concept of the arrow of time to characterize brain activity. Building on this principle, the level of non‐reversibility is computed as follows: first, generate the reversed time series (Figure 2a) and shift the forward and reversed time series in time (Figure 2b). The correlation between time series and their time‐shifted version captures the strength of time dependencies (Figure 2d). The difference between this correlation for the forward signal and the reversed signal captures the asymmetry in the flow of events. For the whole‐brain multidimensional time series, this is captured by the least squares distance between the forward time‐shifted correlation matrix and the reverse time‐shifted correlation matrix (Figure 2f,g). In this study, our parcellation has 506 nodes, which means 506^2^ possible pairs of regional time series to calculate non‐reversibility from. Furthermore, pair‐wise non‐reversibility values are extremely right‐skewed in distribution (see Figure S10), which hinders the computation of single‐subject summarized statistics because meaningful information is “washed out” when computing an average value. Therefore, we took the following approach: each time we compared groups (e.g., MSC vs. TSC at resting state condition), we took the indices of the top 10% most relevant pairs according to the strictly standardized mean difference (SSMD) between them and only considered those to calculate a non‐reversibility value for each subject. It is noteworthy to mention that this way of computing non‐reversibility is not compatible with regression or factorial models, as the neural metric we compute is then not invariant across comparisons, and regression‐based approaches require a predefined comparison‐independent variable at the subject level. Nonetheless, this approach is suitable in our case because our study is focused on developmental stage‐specific differences between educational environments rather than assuming a strictly linear relationship with age, and more desirable because it allows us to reduce the noise in our summarized statistics. Furthermore, discriminative features differ between intrinsic and externally driven dynamics, since the two conditions reflect fundamentally different neural regimes. We therefore analyzed them separately, treating them as distinct conceptual questions for interpretability. To calculate the non‐reversibility for each functional network, we also took only the pairs that belonged to the former 10%. Furthermore, we calculated the average non‐reversibility value of each node, averaged across subjects. We chose an optimal value of the time shift Δt = 1, as when we compute the mean autocorrelation function of all signals, we see that it has already decayed sufficiently at this time shift value. We also chose an optimal amount of time points to extract the non‐reversibility levels from *Tmax*  =  360 (the first 3 min of the recording) for two main reasons: to avoid the risk of having our results muddled by students falling asleep in the fMRI machine, which is not uncommon [110], and to ensure we were able to include as many participants as possible (some subjects did not complete the full 6‐m resting‐state acquisition). To test the reliability of the non‐reversibility estimates derived from the selected segments, we computed Spearman correlations between the first and last halves of each run for those subjects who completed them fully (see Table S10).

More precisely, the level of non‐reversibility is captured through the degree of asymmetry obtained by comparing the pairwise causal relationships of the forward and reversed time series. Let us consider two time series *x_i_* (*t*) and *x_j_* (*t*), and their respective reversed versions *x_i_* ^(*r*)^(*t*) and *x_j_* ^(*r*)^(*t*) (see Figure 2a,b illustrations of this reversal principle), which are part of a multi‐dimensional signal *x*(*t*) as in Figure 2e. The causal dependency between two time series is measured through the time‐shifted correlation at a given shift of shift Δ*t*  =  *T*, expressed as its mutual information. In the forward case, this is:

(1) $$FS_{\mathrm{forward},\;\;\;\mathrm{ij}}\;\left(\Delta t\right)=-\frac{1}{2}\log\left(1-{\langle x_{i}\left(t\right),\;x_{j}\left(t+\Delta t\right)\rangle }^{2}\right)$$

And the reverse:

(2) $$FS_{\mathrm{reversal},\;\;\;\mathrm{ij}}\;\left(\Delta t\right)=-\frac{1}{2}\log\left(1-{\langle {x_{i}}^{\left(r\right)}\left(t\right),\;{x_{j}}^{\left(r\right)}\left(t+\Delta t\right)\rangle }^{2}\right)$$

where the subindexes represent each dimension of the multidimensional time series. In this case, we are analyzing multidimensional BOLD fMRI data, so the subindexes represent spatial parcels of the brain. We selected an optimal value of Δ*t*  =  1.

The level of non‐reversibility is given by the quadratic distance between the forward and reversal time‐shifted matrices:

(3) $$I\left(T\right)=∥FS_{forward}\left(T\right)-FS_{reversal}\left(T\right){∥}_{2}\;$$

That is, non‐reversibility level is defined as the mean value of the absolute squares of the difference between *FS_forward_* (*T*) and *FS_reversal_* (*T*) (see Figure 2g). In other words, if we define this difference as a matrix:

(4) $$FS_{diff,\;ij}={\left(FS_{forward,\;ij}\left(T\right)-FS_{reversal,ij}\left(T\right)\right)}^{2}\;$$

The level of non‐reversibility is the mean of all its elements. The number of elements in this matrix *FS_diff_* grows exponentially with the spatial scale of the time series data. Furthermore, most of these elements have derisory non‐reversibility values: if we plot the values of *FS_diff_* as a histogram, it is extremely right‐skewed. This pulls the mean across all these elements toward zero, washing out the interesting effects of the most non‐reversible nodes. To circumvent this when comparing two groups, we averaged the elements of *FS_diff_* across subjects for each group, which yields two matrices, for example: $FS_{diff}^{\left(M\right)}$ for the Montessori group and $FS_{diff}^{\left(T\right)}$ for the traditional group. To compute the difference between those matrices, we used the SSMD, a measure of effect size computed as the mean divided by the standard deviation of a difference between two random values from different groups. For our example:

(5) $$d_{i,j}=\frac{\left|FS_{diff,\;ij}^{\left(M\right)}-\;FS_{diff,\;ij}^{\left(T\right)}\right|}{\sqrt{{\sigma^{2}}_{ij}^{\left(M\right)}+{\sigma^{2}}_{ij}^{\left(T\right)}}}\;$$

where ${\sigma^{2}}_{ij}^{\left(M\right)}$ and ${\sigma^{2}}_{ij}^{\left(T\right)}$ are the variances of the elements of *FS_diff_* across each group, respectively. We took the indices of the 10% most distant nodes between the group averages according to the SSMD and used only those to average across each subject's *FS_diff_* to obtain a non‐reversibility value, both globally and within each network. To ensure the robustness of this approach, we also computed the same metrics with the top 5%, top 15%, and top 20% nodes (see Figure S12 and Tables S13–S18).

To calculate a non‐reversibility value for each node of the parcellation, we took the sum of the mean across rows and the mean across columns of the matrix *FS_diff_*.

### 4.7 Statistical Analysis

To assess group differences in demographic and background variables, we applied a principled multi‐step strategy. Continuous variables were first tested for normality using the Shapiro‐Wilk test. When the normality assumption was met, group differences were examined with independent‐samples Student's *t*‐tests. For variables violating normality (Shapiro‐Wilk *p* < 0.05), we used Mann‐Whitney U tests, which do not rely on distributional assumptions and provide a robust non‐parametric alternative. Categorical variables were analyzed with chi‐square tests to compare the proportion of MSC and TSC students in each category. This combination of parametric and non‐parametric procedures ensures that each variable was tested using the most statistically appropriate method, maximizing sensitivity while preserving Type I error control.

For the cognitive/affective battery (academic outcomes, creativity, epistemic emotions, and ERQ reappraisal), we first ran a MANCOVA including the six variables as a joint outcome and pedagogy (TSC vs. MSC) and age group (<8.5 vs. ≥8.5 years) as between‐subject factors, with SES and fluid intelligence entered as covariates (Pillai's trace, Type III sums of squares). Significant multivariate effects were followed by univariate ANCOVAs of identical form for each outcome.

Potential effects of head motion were assessed by comparing mean framewise displacement and DVARS across groups using ANOVA, including pedagogy, age group, and gender as factors.

For neural measures derived from the INSIDEOUT framework, statistical significance was assessed using Monte Carlo permutation tests (10000 permutations), in which group labels were randomly shuffled across participants. The test statistic was the difference in group medians. For each comparison, false discovery rate correction was applied across networks using the Benjamini‐Hochberg procedure.

All tests were two‐sided, and statistical significance was set at *p* < 0.05.

## Funding

E. del Agua was supported by the FI Grant (no. 2022FI_B 00337) funded by the Catalan Agency for Management of University and Research Grants (AGAUR). A. Escrichs was supported by the project eBRAIN‐Health – Actionable Multilevel Health Data (id 101058516), funded by the EU Horizon Europe and also by the Grant PID2022‐136216NB‐I00 funded by MICIU/AEI/10.13039/501100011033 and by “ERDF A way of making Europe”, ERDF, EU. Y.S.P is supported by Project NEurological MEchanismS of Injury, and Sleep‐like cellular dynamics (NEMESIS) (ref. 101071900) funded by the EU ERC Synergy Horizon Europe. M.L. Kringelbach is supported by the Centre for Eudaimonia and Human Flourishing (funded by the Pettit and Carlsberg Foundations) and Center for Music in the Brain (funded by the Danish National Research Foundation, DNRF117). S. Denervaud is supported by the Société Académique Vaudoise. G. Deco is supported by Grant PID2022‐136216NB‐I00 funded by MICIU/AEI/10.13039/501100011033 and by “ERDF A way of making Europe”, ERDF, EU, Project NEurological MEchanismS of Injury, and Sleep‐like cellular dynamics (NEMESIS) (ref. 101071900) funded by the EU ERC Synergy Horizon Europe, and AGAUR research support grant (ref. 2021 SGR 00917) funded by the Department of Research and Universities of the Generalitat of Catalunya. A. Diamond was supported by a Canada Research Chair Tier 1 (CRC #950‐27472) from the Canada Excellence Research Chairs Program.

## Conflicts of Interest

The authors declare no conflicts of interest.

## Supporting information




**Supporting File**: advs75400‐sup‐0001‐SuppMat.docx.

## Data Availability

The data that support the findings of this study are available from the corresponding author upon reasonable request.
