# Supplementary material for: Schooling Trajectories and the Development of Brain Dynamics: A Comparative Study of Montessori and Traditional Education
Source: Adv Sci (Weinh). 2026 Apr 20;13(40):e24343. doi: 10.1002/advs.202524343 (PMC13335613; doi:10.1002/advs.202524343)
Supplement: Supplementary file 1 — Supporting File: advs75400‐sup‐0001‐SuppMat.docx. [file ADVS-13-e24343-s001.docx]

Supporting Information

Schooling Trajectories and the Development of Brain Dynamics: A Comparative Study of Montessori and Traditional Education

Elvira del Agua*, Anira Escrichs, Yonatan Sanz Perl, Morten L. Kringelbach, Adele Diamond, Solange Denervaud and Gustavo Deco

**
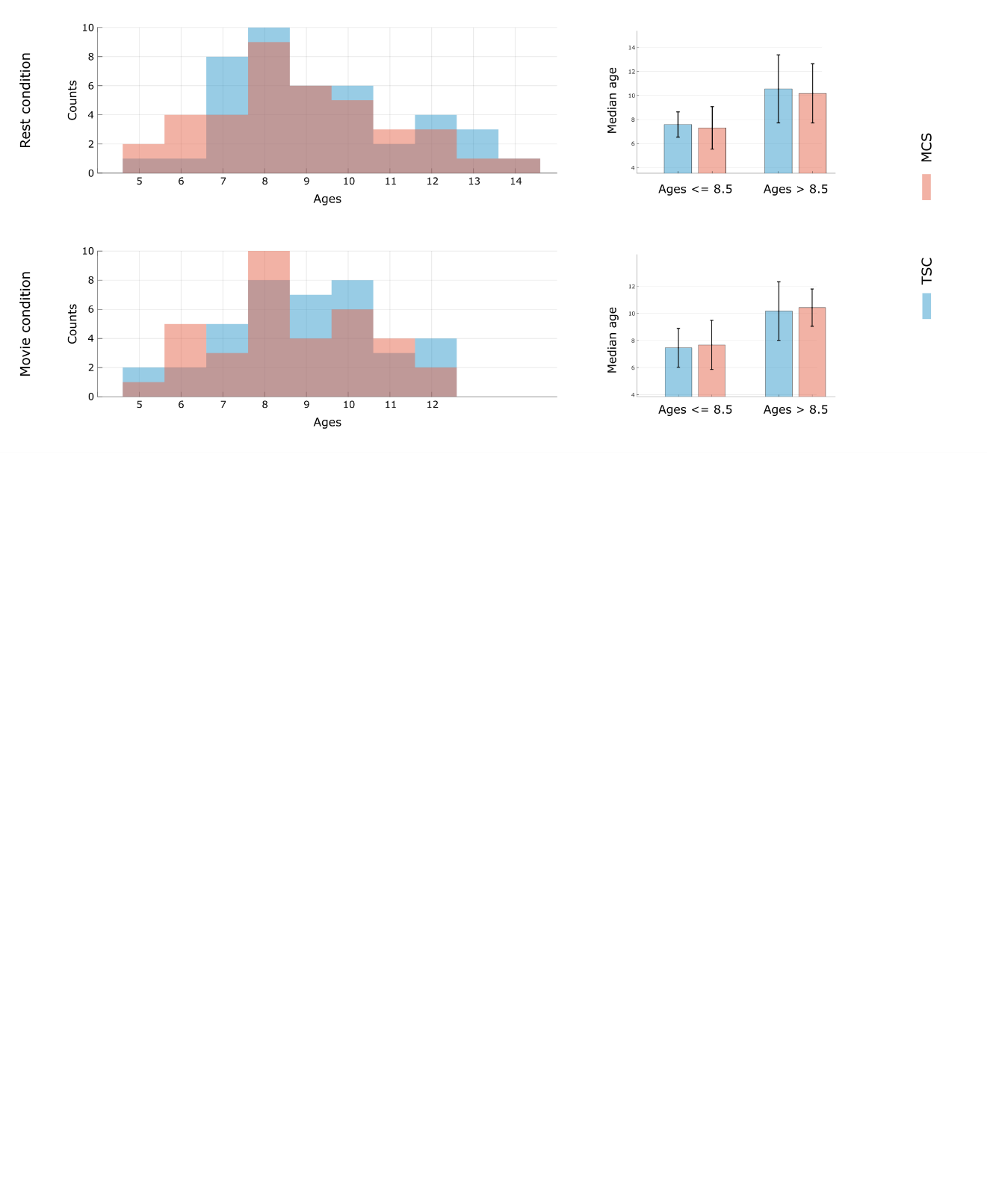
**

**Figure S1.** Distribution of ages of children included in the analysis, in rest condition (top row) and movie condition (bottom row).


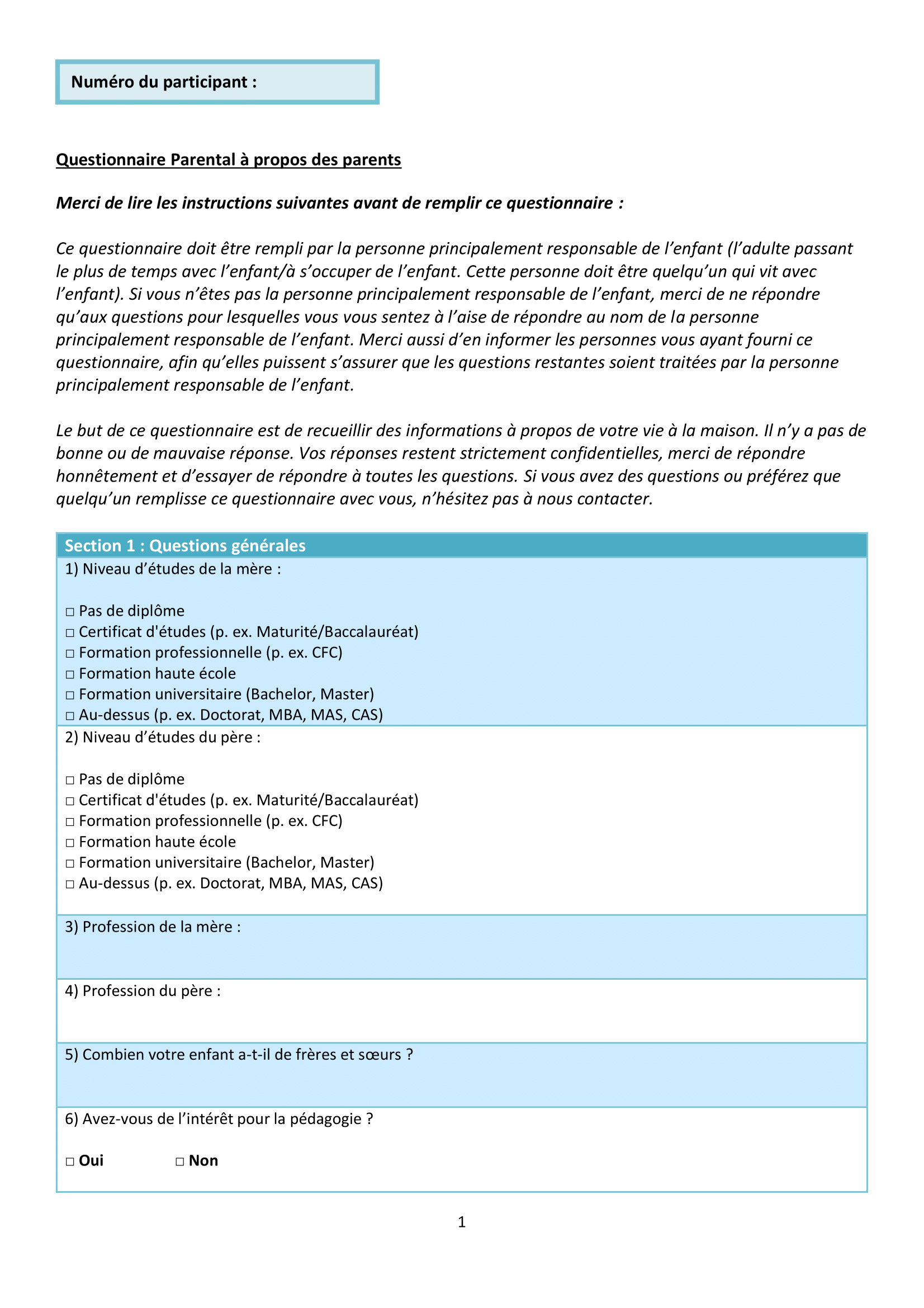


**Figure S2.** Parent questionnaire (page 1).


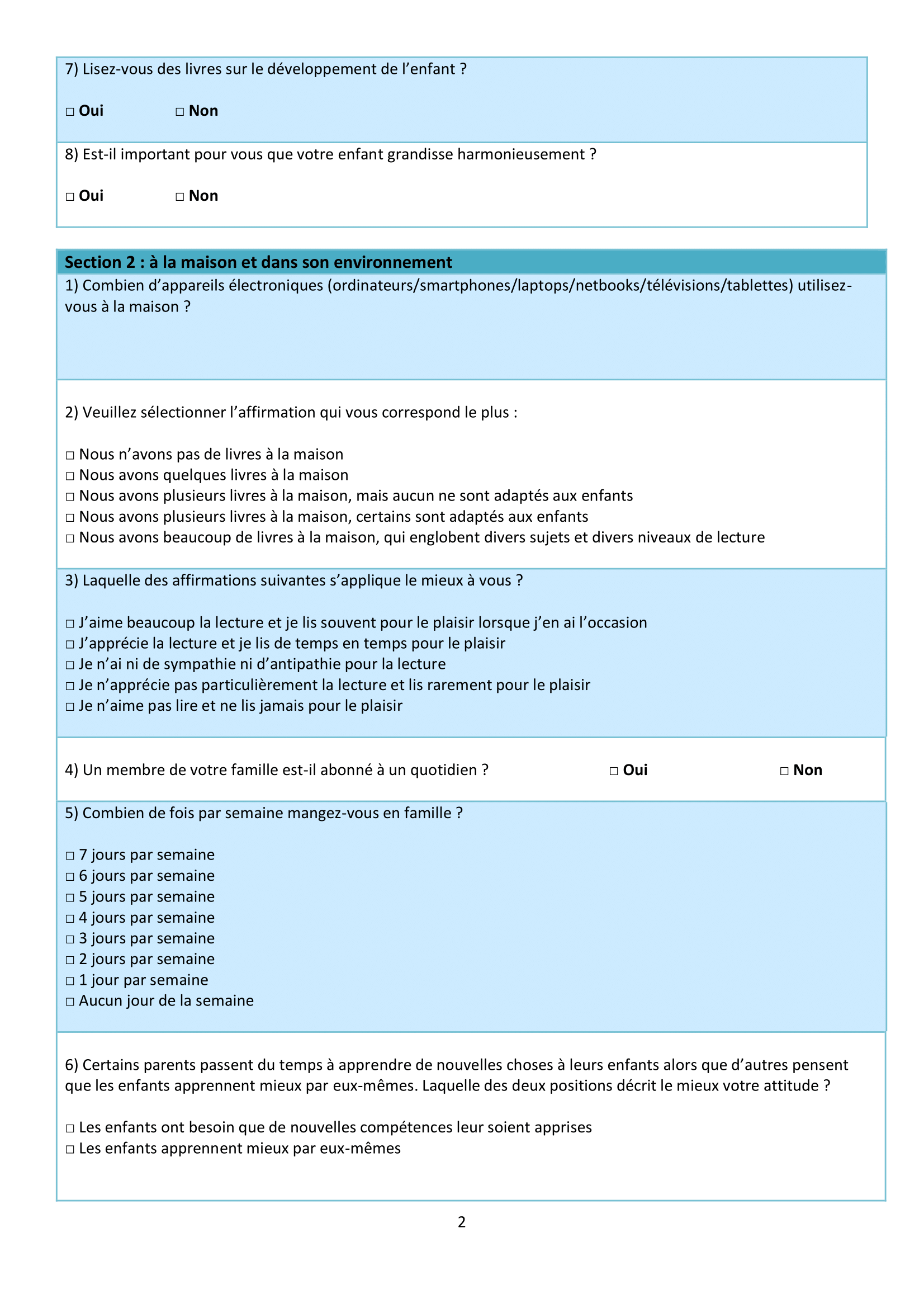


**Figure S3.** Parent questionnaire (page 2).


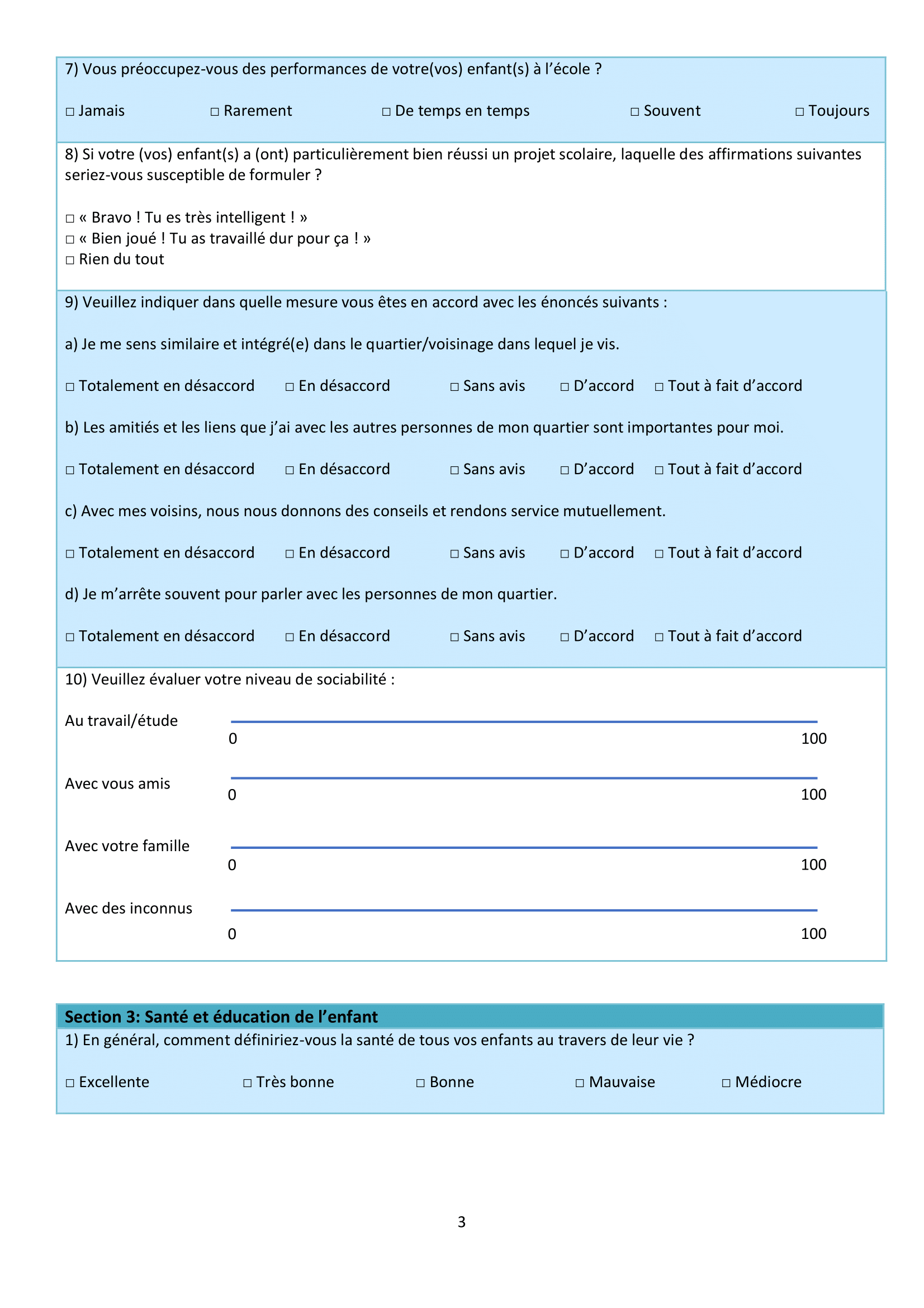


**Figure S4.** Parent questionnaire (page 3).


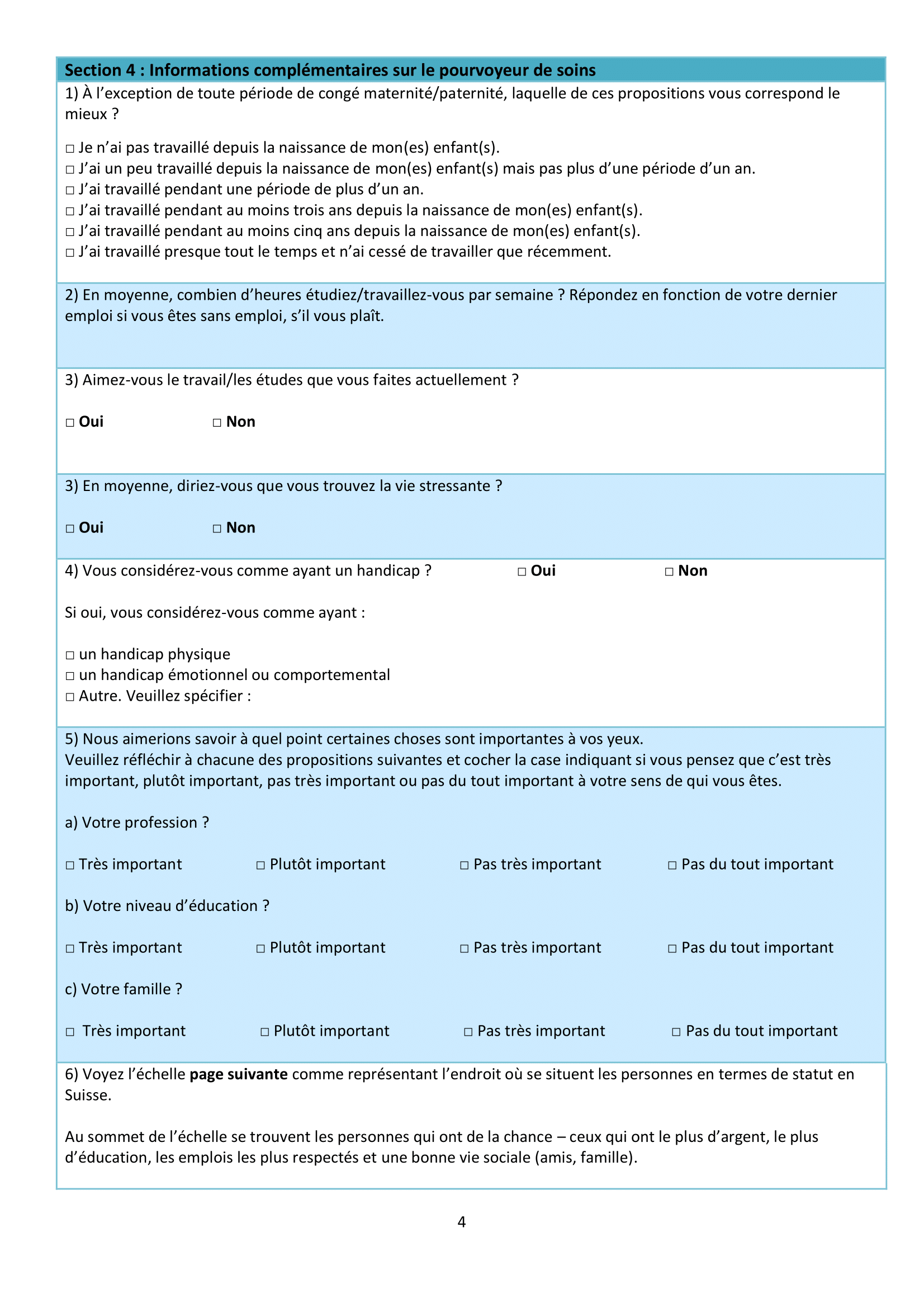


**Figure S5.** Parent questionnaire (page 4).


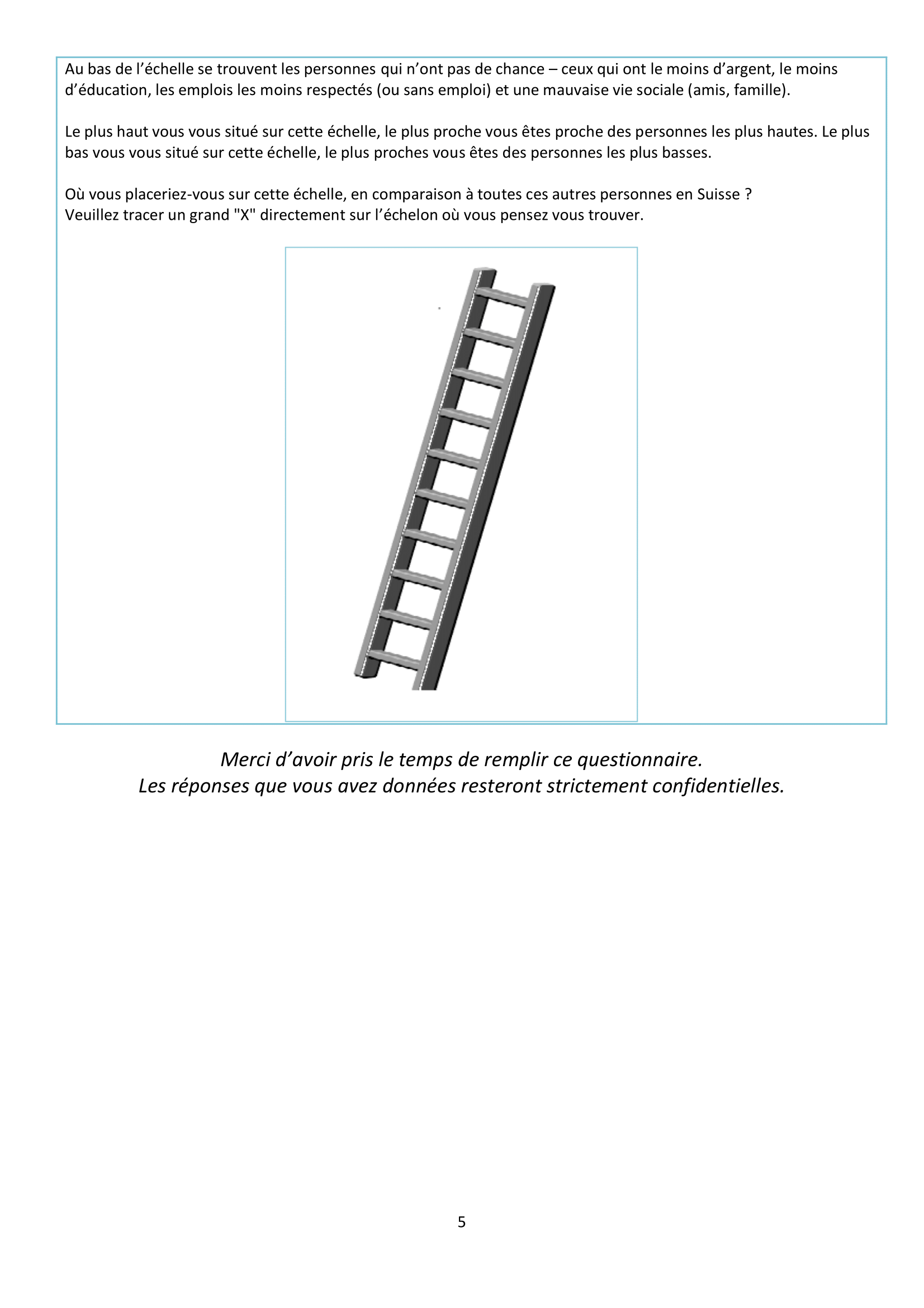


**Figure S6.** Parent questionnaire (page 5).


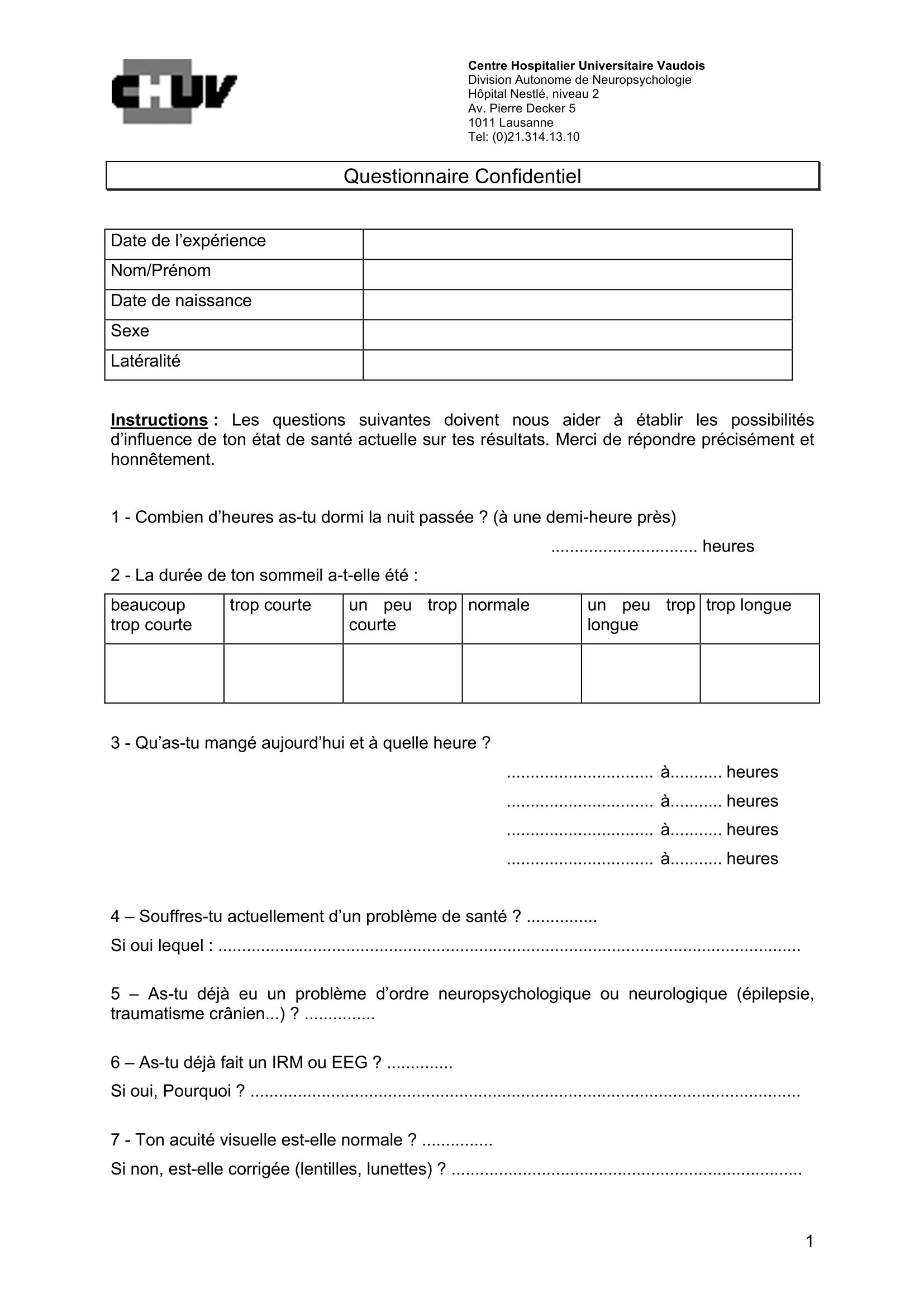


**Figure S7.** Children questionnaire (page 1).


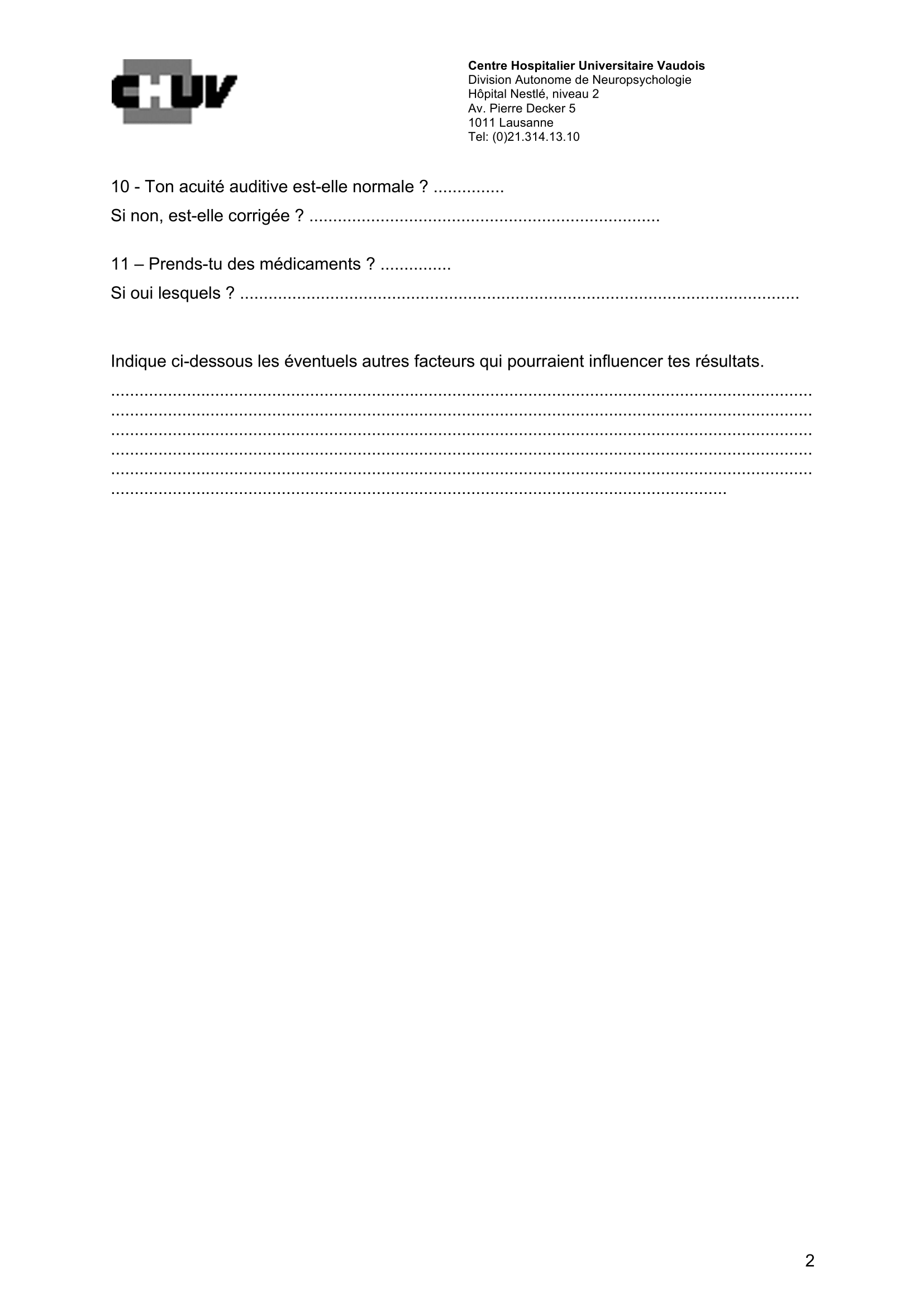


**Figure S8.** Children questionnaire (page 2).


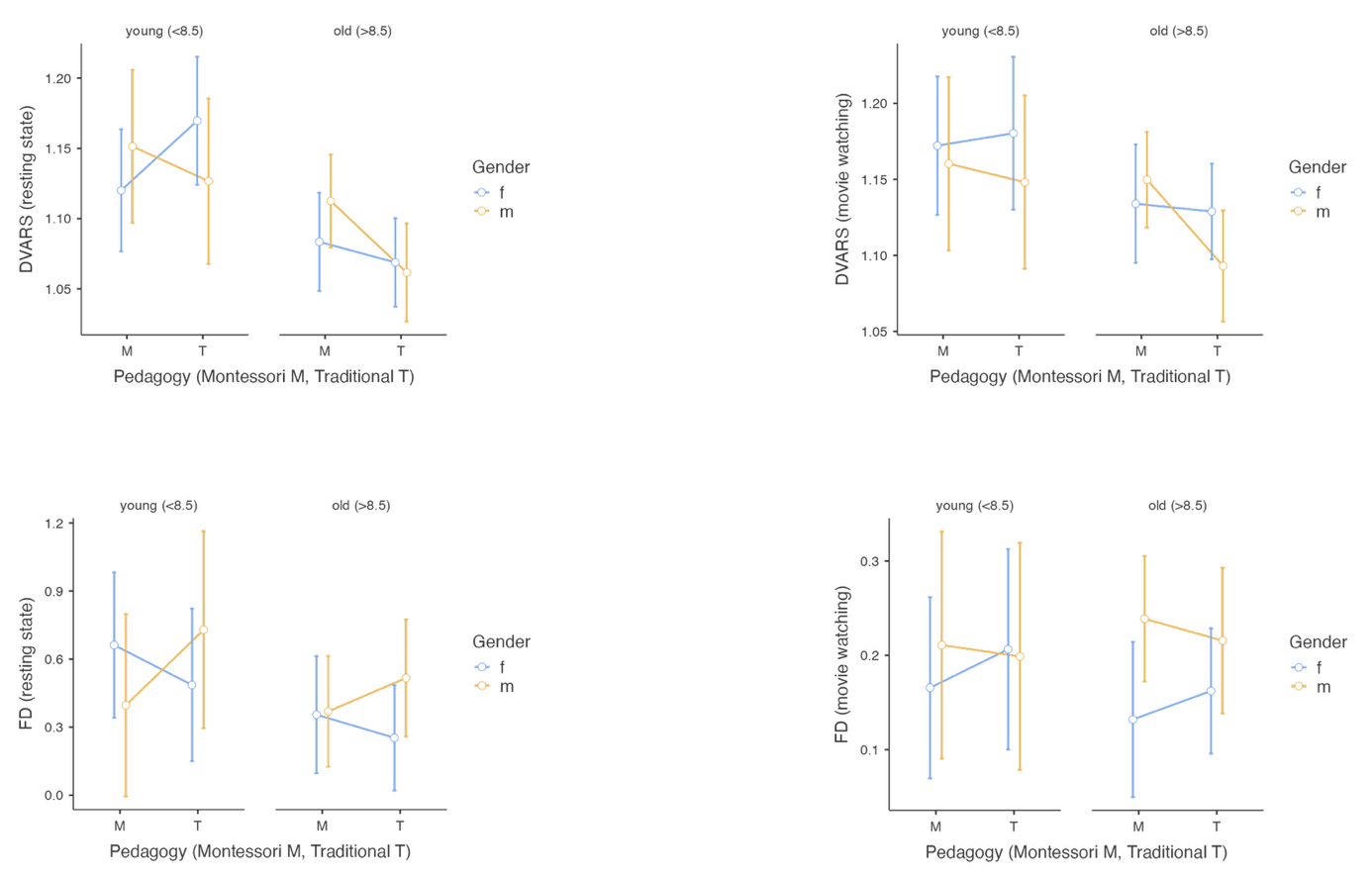


**Figure S9.** FD and DVARS values per age-group, gender, and pedagogical background.


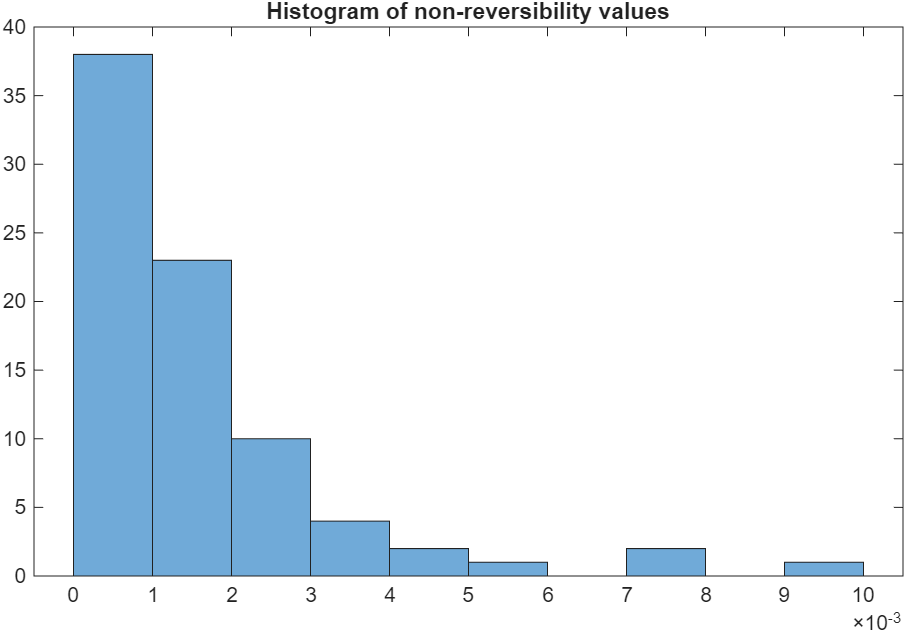


**Figure S10.** Distribution of pair-wise non-reversibility values.

**Table S11.** Test-retest Spearman correlations between first and second half of the neuroimaging run.

|  | Resting state | | Movie Watching | |
| --- | --- | --- | --- | --- |
| Network | r | p | r | p |
| Whole brain | 0.566 | <0.0001 | 0.681 | <0.0001 |
| Subcortical | 0.513 | <0.0001 | 0.679 | <0.0001 |
| Visual | 0.415 | 0.0002 | 0.610 | <0.0001 |
| Somatomotor | 0.532 | <0.0001 | 0.604 | <0.0001 |
| Dorsal Attention | 0.472 | <0.0001 | 0.450 | 0.0001 |
| Ventral Attention | 0.543 | <0.0001 | 0.631 | <0.0001 |
| Limbic | 0.468 | <0.0001 | 0.565 | <0.0001 |
| Frontoparietal | 0.410 | 0.0003 | 0.551 | <0.0001 |
| Default Mode | 0.489 | <0.0001 | 0.472 | <0.0001 |


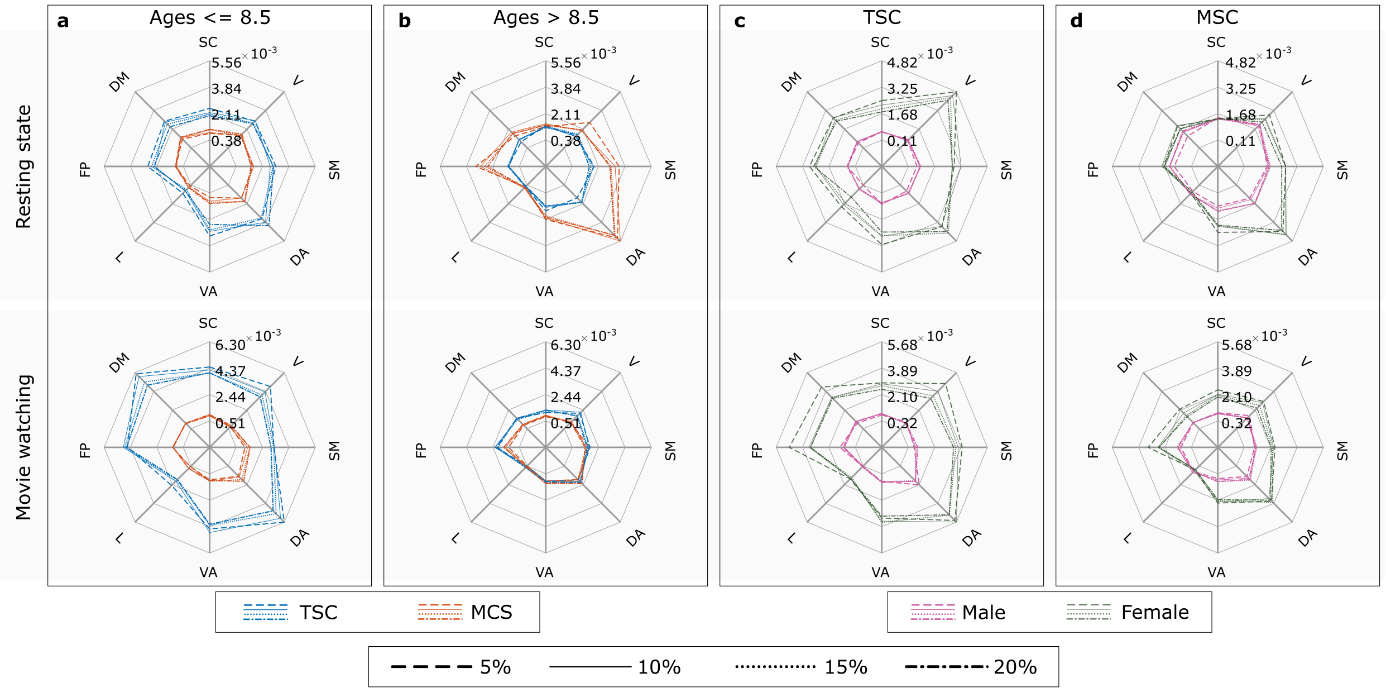


**Figure S12. Using different thresholds for the top percentage of nodes to compute non-reversibility from.** To explore the robustness of our results based on the choice of using the top 10% most important nodes within each comparison, we also computed non-reversibility metrics using the 5%, 15% and 20% top nodes. In this figure we show these results at the resting-state network level (Subcortical (SC), Visual (V), Sensorimotor (SM), Dorsal Attention (DA), Ventral Attention (VA), Limbic (L), Frontoparietal (FP), and Default Mode (DM)) for each of the main comparisons discussed in the manuscript: **a)** Younger group (ages <= 8.5) of traditionally schooled children (TSC) versus Montessori-schooled children (MSC), at rest (top row) and at movie-watching condition (bottom row). **b)** Older group (ages > 8.5) of TSC vs MSC, at rest (top row) and at movie-watching condition (bottom row). **c)** Males vs. females comparison within the TSC group, at rest (top row) and at movie-watching condition (bottom row). **d)** Males vs. females comparison within the MSC group, at rest (top row) and at movie-watching condition (bottom row).

**Table S13**. Main p-values and effect sizes for the comparisons in this study, at the threshold of 5% top nodes.

| Comparison | Network | Resting state | | Movie watching | |
| --- | --- | --- | --- | --- | --- |
|  |  | p | SSDN | p | SSDN |
| TSC vs MSC (Ages <= 8.5) | Whole brain | <0.0001 | 0.895 | <0.0001 | 1.304 |
|  | Subcortical | 0.0002 | 0.757 | <0.0001 | 1.150 |
|  | Visual | 0.0083 | 0.704 | <0.0001 | 1.094 |
|  | Somatomotor | 0.0061 | 0.700 | 0.0038 | 0.788 |
|  | Dorsal Attention | 0.0489 | 0.588 | <0.0001 | 0.975 |
|  | Ventral Attention | <0.0001 | 0.961 | <0.0001 | 1.026 |
|  | Limbic | 0.0048 | 0.642 | <0.0001 | 1.243 |
|  | Frontoparietal | 0.0002 | 0.928 | <0.0001 | 1.228 |
|  | Default mode | 0.0006 | 0.861 | <0.0001 | 1.518 |
| TSC vs MSC (Ages > 8.5) | Whole brain | 0.0123 | -0.744 | 0.2898 | 0.156 |
|  | Subcortical | 0.4027 | 0.299 | 0.0804 | 0.551 |
|  | Visual | 0.0271 | -0.595 | 0.0016 | 0.666 |
|  | Somatomotor | 0.0076 | -0.636 | 0.3687 | -0.189 |
|  | Dorsal Attention | 0.0010 | -0.786 | 0.4305 | 0.036 |
|  | Ventral Attention | 0.2508 | -0.194 | 0.3925 | -0.129 |
|  | Limbic | 0.4637 | -0.118 | 0.4363 | 0.312 |
|  | Frontoparietal | 0.0025 | -0.664 | 0.0719 | 0.483 |
|  | Default Mode | 0.0735 | -0.402 | 0.0103 | 0.633 |
| Males vs Females  (TSC) | Whole brain | <0.0001 | -1.174 | <0.0001 | -1.217 |
|  | Subcortical | <0.0001 | -1.014 | <0.0001 | -1.033 |
|  | Visual | <0.0001 | -1.147 | <0.0001 | -1.160 |
|  | Somatomotor | <0.0001 | -1.014 | <0.0001 | -1.072 |
|  | Dorsal Attention | <0.0001 | -0.974 | <0.0001 | -0.804 |
|  | Ventral Attention | <0.0001 | -0.958 | 0.0002 | -1.075 |
|  | Limbic | <0.0001 | -1.040 | <0.0001 | -1.006 |
|  | Frontoparietal | <0.0001 | -0.983 | <0.0001 | -1.093 |
|  | Default Mode | <0.0001 | -1.193 | <0.0001 | -1.291 |
| Males vs Females  (MSC) | Whole brain | 0.0007 | -0.753 | <0.0001 | -1.059 |
|  | Subcortical | 0.3523 | -0.197 | <0.0001 | -1.023 |
|  | Visual | 0.1398 | -0.334 | 0.0093 | -0.759 |
|  | Somatomotor | 0.0004 | -0.698 | 0.0029 | -0.627 |
|  | Dorsal Attention | 0.0005 | -0.846 | 0.0002 | -1.105 |
|  | Ventral Attention | 0.0001 | -0.915 | <0.0001 | -0.947 |
|  | Limbic | 0.2645 | -0.226 | 0.1644 | 0.275 |
|  | Frontoparietal | 0.0052 | -0.713 | <0.0001 | -1.231 |
|  | Default Mode | 0.0030 | -0.594 | 0.0002 | -1.007 |

**Table S14**. Main p-values and effect sizes for the comparisons in this study, at the threshold of 15% top nodes.

| Comparison | Network | Resting state | | Movie watching | |
| --- | --- | --- | --- | --- | --- |
|  |  | p | SSDN | p | SSDN |
| TSC vs MSC (Ages <= 8.5) | Whole brain | 0.0007 | 0.738 | <0.0001 | 1.010 |
|  | Subcortical | 0.0005 | 0.691 | <0.0001 | 1.011 |
|  | Visual | 0.0286 | 0.622 | <0.0001 | 0.899 |
|  | Somatomotor | 0.0224 | 0.613 | 0.0172 | 0.628 |
|  | Dorsal Attention | 0.0352 | 0.562 | <0.0001 | 0.932 |
|  | Ventral Attention | 0.0004 | 0.813 | 0.0004 | 0.866 |
|  | Limbic | 0.0792 | 0.454 | 0.0002 | 1.076 |
|  | Frontoparietal | 0.0015 | 0.802 | <0.0001 | 1.013 |
|  | Default mode | 0.0083 | 0.610 | <0.0001 | 1.239 |
| TSC vs MSC (Ages > 8.5) | Whole brain | 0.0554 | -0.538 | 0.253 | 0.209 |
|  | Subcortical | 0.4183 | 0.306 | 0.0563 | 0.509 |
|  | Visual | 0.1469 | -0.502 | 0.0275 | 0.543 |
|  | Somatomotor | 0.0683 | -0.268 | 0.3708 | -0.184 |
|  | Dorsal Attention | 0.0082 | -0.549 | 0.4419 | 0.086 |
|  | Ventral Attention | 0.1252 | 0.058 | 0.4371 | -0.051 |
|  | Limbic | 0.3975 | 0.215 | 0.1321 | 0.285 |
|  | Frontoparietal | 0.0036 | -0.481 | 0.1087 | 0.407 |
|  | Default Mode | 0.1787 | -0.284 | 0.0242 | 0.580 |
| Males vs Females  (TSC) | Whole brain | <0.0001 | -0.979 | <0.0001 | -1.052 |
|  | Subcortical | <0.0001 | -0.872 | 0.0003 | -0.962 |
|  | Visual | <0.0001 | -0.990 | <0.0001 | -0.971 |
|  | Somatomotor | <0.0001 | -0.851 | <0.0001 | -0.955 |
|  | Dorsal Attention | <0.0001 | -0.839 | <0.0001 | -0.780 |
|  | Ventral Attention | <0.0001 | -0.849 | 0.0002 | -0.922 |
|  | Limbic | 0.0002 | -0.777 | <0.0001 | -0.938 |
|  | Frontoparietal | <0.0001 | -0.910 | <0.0001 | -0.961 |
|  | Default Mode | <0.0001 | -1.032 | <0.0001 | -1.074 |
| Males vs Females  (MSC) | Whole brain | 0.0277 | -0.505 | 0.0045 | -0.651 |
|  | Subcortical | 0.4413 | -0.248 | 0.0002 | -0.718 |
|  | Visual | 0.3318 | -0.136 | 0.0094 | -0.590 |
|  | Somatomotor | 0.0044 | -0.598 | 0.0016 | -0.301 |
|  | Dorsal Attention | 0.0010 | -0.866 | <0.0001 | -0.902 |
|  | Ventral Attention | 0.0013 | -0.645 | 0.0003 | -0.686 |
|  | Limbic | 0.1677 | -0.190 | 0.2648 | -0.028 |
|  | Frontoparietal | 0.0865 | -0.535 | 0.0009 | -0.857 |
|  | Default Mode | 0.1155 | -0.356 | 0.0023 | -0.739 |

**Table S15**. Main p-values and effect sizes for the comparisons in this study, at the threshold of 20% top nodes.

| Comparison | Network | Resting state | | Movie watching | |
| --- | --- | --- | --- | --- | --- |
|  |  | p | SSDN | p | SSDN |
| TSC vs MSC (Ages <= 8.5) | Whole brain | 0.0028 | 0.687 | 0.0002 | 1.035 |
|  | Subcortical | 0.0010 | 0.652 | 0.0005 | 0.957 |
|  | Visual | 0.0438 | 0.573 | <0.0001 | 0.862 |
|  | Somatomotor | 0.0215 | 0.598 | 0.0410 | 0.557 |
|  | Dorsal Attention | 0.0245 | 0.539 | <0.0001 | 0.863 |
|  | Ventral Attention | 0.0101 | 0.758 | 0.0004 | 0.841 |
|  | Limbic | 0.0742 | 0.480 | <0.0001 | 0.937 |
|  | Frontoparietal | 0.0027 | 0.754 | <0.0001 | 0.965 |
|  | Default mode | 0.0183 | 0.614 | 0.0002 | 1.150 |
| TSC vs MSC (Ages > 8.5) | Whole brain | 0.0613 | -0.479 | 0.2735 | 0.200 |
|  | Subcortical | 0.3805 | 0.313 | 0.0274 | 0.493 |
|  | Visual | 0.1327 | -0.482 | 0.0440 | 0.461 |
|  | Somatomotor | 0.0505 | -0.258 | 0.4087 | -0.168 |
|  | Dorsal Attention | 0.0075 | -0.500 | 0.4413 | -0.126 |
|  | Ventral Attention | 0.0893 | 0.078 | 0.4368 | -0.089 |
|  | Limbic | 0.3555 | 0.023 | 0.1756 | 0.331 |
|  | Frontoparietal | 0.0252 | -0.467 | 0.2312 | 0.365 |
|  | Default Mode | 0.1923 | -0.283 | 0.0147 | 0.524 |
| Males vs Females  (TSC) | Whole brain | <0.0001 | -0.919 | <0.0001 | -1.001 |
|  | Subcortical | <0.0001 | -0.819 | 0.0010 | -0.921 |
|  | Visual | <0.0001 | -0.940 | <0.0001 | -0.933 |
|  | Somatomotor | <0.0001 | -0.768 | <0.0001 | -0.917 |
|  | Dorsal Attention | <0.0001 | -0.786 | <0.0001 | -0.742 |
|  | Ventral Attention | <0.0001 | -0.790 | <0.0001 | -0.889 |
|  | Limbic | 0.0019 | -0.636 | <0.0001 | -0.911 |
|  | Frontoparietal | <0.0001 | -0.885 | 0.0004 | -0.929 |
|  | Default Mode | <0.0001 | -0.998 | <0.0001 | -1.054 |
| Males vs Females  (MSC) | Whole brain | 0.0464 | -0.421 | 0.0061 | -0.547 |
|  | Subcortical | 0.4333 | -0.232 | 0.0003 | -0.614 |
|  | Visual | 0.4268 | -0.046 | 0.0160 | -0.528 |
|  | Somatomotor | 0.0165 | -0.534 | 0.0027 | -0.219 |
|  | Dorsal Attention | 0.0025 | -0.805 | 0.0002 | -0.868 |
|  | Ventral Attention | 0.0056 | -0.580 | 0.0007 | -0.649 |
|  | Limbic | 0.1298 | -0.042 | 0.2742 | -0.076 |
|  | Frontoparietal | 0.1181 | -0.535 | 0.0013 | -0.737 |
|  | Default Mode | 0.1516 | -0.307 | 0.0066 | -0.631 |

**Table S16**. Correlation between irreversibility values of each group at the 5% threshold of top nodes and the 10% threshold of top nodes

|  | Rest | | Movie | |
| --- | --- | --- | --- | --- |
|  | TSC | MSC | TSC | MSC |
| Ages <= 8.5 | 0.9987 | 0.9934 | 0.9965 | 0.9978 |
| Ages > 8.5 | 0.9971 | 0.9986 | 0.9963 | 0.9980 |
| Male | 0.9967 | 0.9916 | 0.9952 | 0.9848 |
| Female | 0.9978 | 0.9989 | 0.9980 | 0.9949 |

**Table S17**. Correlation between irreversibility values of each group at the 15% threshold of top nodes and the 10% threshold of top nodes

|  | Rest | | Movie | |
| --- | --- | --- | --- | --- |
|  | TSC | MSC | TSC | MSC |
| Ages <= 8.5 | 0.9990 | 0.9974 | 0.9986 | 0.9991 |
| Ages > 8.5 | 0.9993 | 0.9989 | 0.9989 | 0.9981 |
| Male | 0.9991 | 0.9947 | 0.9982 | 0.9974 |
| Female | 0.9993 | 0.9996 | 0.9991 | 0.9986 |

**Table S18**. Correlation between irreversibility values of each group at the 20% threshold of top nodes and the 10% threshold of top nodes

|  | Rest | | Movie | |
| --- | --- | --- | --- | --- |
|  | TSC | MSC | TSC | MSC |
| Ages <= 8.5 | 0.9956 | 0.9882 | 0.9957 | 0.9971 |
| Ages > 8.5 | 0.9985 | 0.9965 | 0.9964 | 0.9939 |
| Male | 0.9974 | 0.9898 | 0.9960 | 0.9935 |
| Female | 0.9976 | 0.9988 | 0.9976 | 0.9941 |
